# Few sex differences in regional gray matter volume growth trajectories across early childhood

**DOI:** 10.1162/imag_a_00154

**Published:** 2024-05-20

**Authors:** Madison Long, Curtis Ostertag, Jess E. Reynolds, Jing Zheng, Bennett Landman, Yuankai Huo, Nils D. Forkert, Catherine Lebel

**Affiliations:** Department of Radiology, University of Calgary, Calgary, Canada; Cummings School of Medicine, University of Calgary, Calgary, Canada; Owerko Centre, Alberta Children Hospital Research Institute, University of Calgary, Calgary, Canada; Hotchkiss Brain Institute, University of Calgary, Calgary, Canada; Telethon Kids Institute, The University of Western Australia, Perth, Australia; Department of Electrical and Computer Engineering, Vanderbilt University, Nashville, TN, United States; Department of Computer Science, Vanderbilt University, Nashville, TN, United States

**Keywords:** brain development, structural MRI, gray matter volume, development trajectories, early childhood, sex differences

## Abstract

Sex-specific developmental differences in brain structure have been documented in older children and adolescents, with females generally showing smaller overall brain volumes and earlier peak ages than males. However, sex differences in gray matter structural development in early childhood are less studied. We characterized sex-specific trajectories of gray matter volume development in children aged 2–8 years. We acquired anatomical magnetic resonance imaging (MRI) of the brain at the Alberta Children's Hospital in 123 typically developing children. Most children were scanned multiple times, for a total of 393 scans (mean = 3.2 scans/subject). We segmented T1-weighted structural MRI with MaCRUISE to define 116 regions and measured both absolute volumes (mm^3^) and proportional volumes (percent of intracranial volume). We characterized growth trajectories of gray matter volume for these brain regions between 2 and 8 years using mixed-effects models, showing volume increases, with most posterior and temporo-parietal regions peaking before 8 years. We found widespread main effects of sex, with males having larger volumes in 86% of brain regions. However, there were no significant sex differences in trajectories (age or age^2^terms) for absolute volume. Proportional volumes of the right occipital fusiform gyrus and left medial postcentral gyrus showed significant age-by-sex interactions where females had steeper volume decreases than males. This study also confirms regional patterns observed in previous studies of older children, such as posterior-to-anterior timing of brain maturation. These results provide a comprehensive picture of gray matter volume development across early childhood, and suggest that sex differences do not emerge until later in development.

## 1 Introduction

Early childhood is a critical period of brain and cognitive development (Thomas & Johnson, 2008). Cross-sectional and longitudinal neuroimaging evidence in healthy humans has shown that from birth through adulthood, changes in whole-brain gray matter volume follow a non-linear trajectory with rapid increases in early life followed by gradual decreases during adolescence (Bethlehem et al., 2022;Brown et al., 2012;Lenroot et al., 2007;Mills et al., 2016;Narvacan et al., 2017;Remer et al., 2017;Rutherford et al., 2022). Increases in gray matter volume observed via magnetic resonance imaging (MRI) are thought to reflect biological processes such as neural proliferation, dendritic spine arborization, synaptogenesis, gliogenesis and maturation, maturation of the neuropil, and gyrification, while decreases are historically thought to reflect synaptic pruning and myelination, resulting in a change in the gray to white matter ratio (Deoni et al., 2015;Giedd & Rapoport, 2010;Raznahan et al., 2011;Remer et al., 2017). Gray matter maturation is spatiotemporally variable: parietal and inferior regions of the cortex reach peak volumes slightly earlier than frontal, temporal, superior, and subcortical regions (Bethlehem et al., 2022;Lenroot & Giedd, 2006;Lenroot et al., 2007;Tanaka et al., 2012;Uematsu et al., 2012;Zhang et al., 2021). However, only a few neuroimaging studies have been conducted spanning early childhood, which is a critical development period. Furthermore, there is a lack of evidence on brain development in early childhood from longitudinal studies, which are necessary for capturing true development trajectories (Di Biase et al., 2023). A more thorough understanding of typical gray matter development patterns in early childhood would help provide a basis for identifying atypical trajectories.

Sex differences in brain volumes have been widely reported in previous research (Cosgrove et al., 2007;De Bellis et al., 2001;DeCasien et al., 2022;Gennatas et al., 2017;Giedd et al., 2012;Goldstein et al., 2001;Gur et al., 2002;Kaczkurkin et al., 2019;Lenroot & Giedd, 2010;Lenroot et al., 2007;Lotze et al., 2019;Sanchis-Segura et al., 2019;Tanaka et al., 2012), which robustly show that males, on average, have larger gray matter, white matter, and total brain volumes than females across the lifespan. Previous research has also indicated that males have a more protracted development trajectory than females across late childhood and adolescence (De Bellis et al., 2001;Giedd et al., 2009;Lenroot et al., 2007;Tanaka et al., 2012). Notably, studies that examined gray matter volume as a proportion of total brain or intracranial volume suggest that females have proportionally more gray matter than males (Goldstein et al., 2001;Gur et al., 2002). Given the high degree of correlation between sex and total brain volume, both raw volume and normalized gray matter volume should be considered when testing for developmental sex differences (Giedd et al., 2012;Mills et al., 2016). While the presence of sex differences in brain structure during adolescence and adulthood is well established, it remains unclear whether those sex differences are already present in early childhood. Examining the timing with which brain sex differences emerge may help explain mechanisms underlying sex-differential prevalence rates for mental health and neurodevelopmental disorders (Copeland et al., 2014;Gogos et al., 2019;Halladay et al., 2015;Mathes et al., 2019;Maughan et al., 2013;Mazure & Swendsen, 2016;Merikangas & Almasy, 2020;Ramtekkar et al., 2010;Rutter et al., 2003;Smink et al., 2012).

The present study aimed to characterize longitudinal changes in regional gray matter volume across early childhood (ages 2–8 years) and compare development trajectories between males and females. We hypothesized that most group-level regional volume trajectories would show a quadratic increase in volume over time with a slowing of growth towards 8 years (inverted-U shaped trajectory). Additionally, we hypothesized that there would be significant effects of sex, both main effects (with males having larger total volumes) and interactions (with males having slower development than females). Regarding proportional volume, we hypothesized that global and regional gray matter volume would decrease over time and that sex effects would diminish when normalizing gray matter volume as a percent of total intracranial volume. While it is increasingly common to report additional gray matter measurements such as cortical thickness and surface area, which are more closely related to cytoarchitectural changes than gray matter volume alone, this manuscript focuses on gray matter volume for several reasons. First, we report development trajectories for both cortical and subcortical regions, the latter of which only volume can be reliably reported. Second, volume has been the most widely reported gray matter metric in previous studies, and thus reporting volume in this novel sample simplifies connecting our results to previous research.

## 2 Materials and Methods

### 2.1 Participants and longitudinal MRI acquisition

**Fig. 1.**
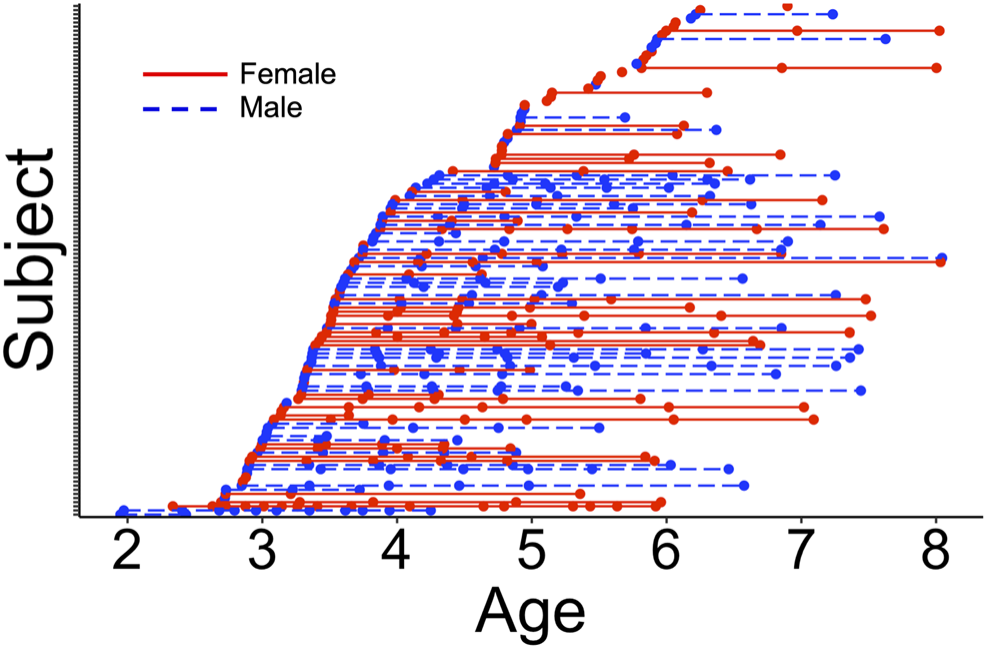
Age at the time of scanning for all subjects included in this sample. Each line represents a subject, and each dot is a single scan session used in the volume analysis.

This study was approved by The University of Calgary Conjoint Health Research Ethics Board (CHREB; REB13-0020). The present sample included 123 children from the prospective longitudinal Calgary Preschool MRI Dataset (Reynolds et al., 2020). The cohort consists of healthy children with no physical, neurological, or psychiatric diagnoses who were born at >35 weeks gestation. Children were recruited to the study between ages 2 and 5 years and completed MRI scans at 6–12 month intervals (N = 393 scans, ~3.2 scans/subject;Fig. 1) for a full age range of 1.97–8.04 years. 90% of parents were married/common law, 84% of families identified as White, 87% of mothers had an undergraduate degree or more, and 70% of family incomes were at or above the median income for the city of Calgary, Alberta, Canada (Table 1). MRI data were collected at the Alberta Children's Hospital on a research-dedicated 3 T GE MR750w MRI scanner with a 32-channel head coil. The MRI protocol included T1-weighted anatomical images (FSPGR BRAVO with 0.9 × 0.9 × 0.9 mm resolution, 210 axial slices, TR = 8.23 ms, TE = 3.76 ms, flip angle = 12 degrees, matrix size = 512 × 512, and inversion time = 540 ms). During T1 acquisition, children were awake viewing a movie of their choice or sleeping naturally.

**Table 1.** Sample demographics by sex.

		Male	Female
Age at session 1	Mean	3.9	4.2
	Median	3.7	3.7
	Min	1.9	2.3
	Max	6.2	6.3
Parent marital status	Single	0	1
	married/common law	56	55
	divorced/separated	3	0
Maternal education	High School Diploma	2	0
	Some postsecondary	1	4
	Trade/Technical Diploma	4	15
	Undergraduate degree	27	25
	sSome post-graduate	1	0
	Graduate degree	0	12
Family income	29,000–49,999	1	1
	50,000–74,999	0	3
	75,000–99,999	8	13
	100,000–124,999	13	8
	125,000–149,999	3	2
	150,000–174,999	8	1
	175,000 and up	25	20
Race	White	51	52
	Asian/pacific Islander	2	1
	Filipino	2	1
	Multiracial	3	1

### 2.2 MRI processing, segmentation, and quality control

Images were initially assessed for quality at the scanner during acquisition; sequences were repeated if necessary and if time permitted. Images were also examined for motion and those with major motion artifacts were excluded. During processing, N4 bias-corrected images (Cox, 1996;Cox & Hyde, 1997) were resampled to a voxel size of 1 mm^3^in preparation for multi-atlas segmentation combined with cortical reconstruction using implicit surface evolution (MaCRUISE;Huo, Carass, et al., 2016;Huo, Plassard, et al., 2016;Huo et al., 2017). MaCRUISE integrates the processes of cortical reconstruction and multi-atlas segmentation to produce reliable and consistent cortical surface parcellations in anatomical agreement with brain segmentations (Huo, Carass, et al., 2016;Huo, Plassard, et al., 2016;Huo et al., 2017).

MaCRUISE has systematically been shown to reduce spatial inconsistencies between volumetric segmentation and surface parcellations in aging adult populations as compared to Freesurfer (Huo, Plassard, et al., 2016). We observed similarly incorrect surfaces in our pediatric data when processed with Freesurfer and subsequently decided to process all T1-weighted images used in this study with MaCRUISE. In the MaCRUISE pipeline (Huo, Plassard, et al., 2016), skull and dura stripped images are subject to both multi-atlas segmentation of 132 regions (Asman & Landman, 2012,^2013^;Bermudez et al., 2020;Klein et al., 2010) and TOpology-preserving Anatomical Segmentation (TOADS) fuzzy membership segmentation (Bazin & Pham, 2008). MaCRUISE then fuses the rigid multi-atlas and TOADS segmentations, resulting in a full cerebrum segmentation comprising gray matter and white matter regions. To achieve a cortical reconstruction consistent with the segmentations, MaCRUISE applies multi-atlas anatomically consistent gray matter enhancement (MaACE;Han & Fischl, 2007;Sethian, 1999) to the gray matter component while applying a topology correction to the white matter component (Han et al., 2001,^2002^). These refined gray and white matter segmentations form the outer and inner surfaces of the reconstructed cortex, respectively. Lastly, to resolve any remaining disagreements between the multi-atlas segmentation and reconstructed surfaces, MaCRUISE refines boundaries in the multi-atlas segmentation using the inner and outer cortical surfaces (Huo, Plassard, et al., 2016). While MaCRUISE is a surface-based volume pipeline (rather than voxel-wise), vertex-wise measurements for cortical thickness and surface area are not readily available as outputs from this pipeline and require further processing, outside the scope of this present study. We extracted the refined segmentations (in mm^3^) for analyses of absolute total gray matter volume and absolute regional volume. We additionally computed proportional volume by dividing each region’s volume by total intracranial volume (ICV) and multiplying by 100, resulting in the regional volume expressed as a percent of ICV.

Trained raters checked each individual segmentation image for accuracy, overlayed in each scan’s native T1-space, and assigned them a quality score of 1 (poor), 2 (unsatisfactory), 3 (satisfactory), and 4 (excellent). Segmentations with a quality score < 3 were manually edited and reintroduced to the MaCRUISE pipeline at the segmentation fusion step, in place of the original rigid multi-atlas segmentation. Edited segmentation outputs were reassessed for quality and included in the analysis if the resulting segmentation obtained a quality score of 3 or 4. Of the 452 successfully acquired T1-weighted images considered for this study, 393 (87%) had sufficient quality of both the T1-weighted image and resulting MaCRUISE segmentation. Of the excluded scans, 20 were the subject’s first session, 15 were the subject’s second session, 3 were the third, 4 were the fourth, 8 were the fifth, 4 were the sixth, and the remaining 4 scans were from the seventh timepoint or later. Only 1 subject was completely excluded from the study due to quality concerns. Spaghetti plots of raw volume measurements over time were visually inspected to ensure plausibility of intra-individual change in volume between scans.

### 2.3 Fitting gray matter volume developmental trajectories

To determine the best fitting polynomial trajectory of development, we successively fit and compared nested mixed-effects models of increasing complexity with subjects allowed a random intercept (R package lme4;Bates et al., 2015). We compared the following three models for each region:

$$\text{Null: }Y_{ij}=B_{0i}+\epsilon_{ij}$$

$$\text{Linear: }Y_{ij}=B_{1}\cdot x_{ij}+B_{0i}+\epsilon_{ij}$$

$$\text{Quadratic: }Y_{ij}=B_{2}\cdot x_{ij}^{\;\;2}+B_{1}\cdot x_{ij}+B_{0i}+\epsilon_{ij}$$

where$Y_{ij}$=*j^th^*volume measurement for the*i^th^*subject,$x_{ij}$= the subject’s age at time of scan,$B_{1}$= coefficient for age,$B_{2}$= coefficient for age^2^,$B_{0i}$= subject-specific y-intercept, and$\epsilon_{ij}$= random error.

We determined the best fitting model for each brain region by selecting the one with the lowest Akaike Information Criterion (AIC;Sakamoto et al., 1986), and Bayesian Information Criterion (BIC;Neath & Cavanaugh, 2012), two loss metrics for model selection which also account for model complexity (Cox & Vladescu, 2023). While both AIC and BIC penalize a model for every additional parameter to reduce the risk of over-fitting, the penalty in BIC is greater. In this study, with a limited set of models compared at once, we deemed the risk of over-fitting to be low; In cases where AIC and BIC disagreed, we preferred the model selected by AIC. In this study, AIC and BIC selected the same model in ~75% of regions. Additionally, we calculated the Akaike weight for each model tested. The Akaike weight is a probability between 0 and 1 that a given model is the best at minimizing Kullbach-Leibler discrepancy among a set of models (Wagenmakers & Farrell, 2004). Akaike weights provide a means to compare the strength of evidence in favor of one model, as compared to another. We fit trajectory models for absolute total gray matter and absolute regional volumes (mm^3^) as well as for proportional total and regional gray matter volume expressed as a percent of ICV. For each best fitting model, we extracted the percent change in volume from baseline to peak, age at peak volume, and predicted volume at ages 2 and 8.5 years. We additionally determined partial eta squared (*η_p_^2^*) for all age effects to examine the magnitude of effect that age/time has on developmental changes in gray matter volume.*η_p_^2^*characterizes the proportion of variance in the dependent variable explained by an independent variable while accounting for other covariates in a model. Magnitude of effect size conveyed by*η_p_^2^*is typically benchmarked as follows: small = 0.0099, medium = 0.0588, and large = 0.1379 (Cohen, 2013). Regions with non-null trajectories were retained for further analysis.

### 2.4 Testing for effects of sex

In a second step, using the best fitting non-null regional trajectories described above (i.e., final model was linear or quadratic), we created mixed-effects models to characterize the effects of child sex on gray matter development.

$$\text{Linear: }Y_{ij}=(B_{4}\cdot S_{ij}+B_{1})\cdot x_{ij}+(B_{3}\cdot S_{ij}+B_{0i})+\epsilon_{ij}$$

$$\text{Quadratic: }Y_{ij}=(B_{5}\cdot S_{ij}+B_{2})\cdot x_{ij}^{\;\;2}+(B_{4}\cdot S_{ij}+B_{1})\cdot x_{ij}+(B_{3}\cdot S_{ij}+B_{0i})+\epsilon_{ij}$$

where$Y_{ij}$=*j^th^*volume measurement for the*i^th^*subject,$x_{ij}$= the subject’s age at time of scan,$S_{ij}$= subject sex (male = 0, female = 1),$B_{1}$= coefficient for age,$B_{2}$= coefficient for age^2^,$B_{0i}$= subject-specific y-intercept,$B_{3}$= coefficient for sex main effect,$B_{4}$= coefficient for age-by-sex interaction,$B_{5}$= coefficient for age^2^-by-sex interaction, and$\epsilon_{ij}$= random error.

To correct for multiple comparisons, we grouped p-values for like model components (i.e., all p-values for age-by-sex interaction term, all p-values for age^2^-by-sex interaction term, and all p-values for main effect of sex) and applied a false discovery rate (FDR;Benjamini & Hochberg, 1995) correction to each group of tests (absolute and proportional volume, main effect or interaction of sex) with a threshold of*q <*0.05. When full models including age- or age^2^-by-sex interactions showed no significant effects of sex (*q*> 0.05), models were reduced (interactions removed, main effect of sex retained) and rerun. We examined residual distribution plots for all reported models to confirm adequacy of model fit.

## 3 Results

### 3.1 Total gray matter volume development

**Fig. 2.**
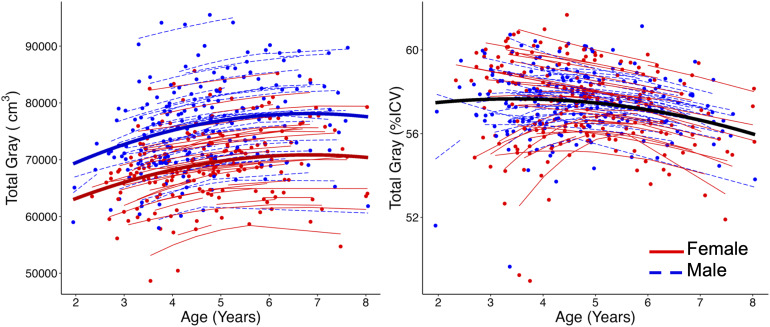
Total gray matter volume showed an absolute increase (left), but a proportional decrease when normalized by intracranial volume (right). Thick lines represent group-level model fit. Thin lines represent best fit lines for individual subjects; each dot is an individual data point. Males had significantly larger absolute total gray matter volume than females, but there were no significant sex by age interaction effects. No sex effects were present for proportional total gray matter volume.

Absolute total gray matter volume had an inverted U-shaped quadratic trajectory, increasing non-linearly by 8% between 2 and 6.85 years, or between 1% and 4% annually (Tables 2, S1;Fig. 2). Proportional gray matter volume was 57.7% of ICV at age 2 and 55.8% of ICV at age 8 years. The trajectory followed a slightly inverted U-shape with a peak at 3.67 years (Table 2, S1;Fig. 2). Males had significantly larger absolute total gray matter volume than females (Table S2;Fig. 2), although this effect disappeared when normalizing by ICV (Table S3,Fig. 2). This pattern was confirmed by evaluation of the AIC values and weights; the best fitting trajectory model for total gray matter volume included a main effect of sex and evidence strongly favored this model (AIC = 9281.99,*w*= 0.84) and proportional volume was best fit by a trajectory including no effects of sex (AIC = 1435.26,*w*= 0.64). We found no significant sex differences in trajectory shape for either absolute or proportional total gray matter volume (Table S2, S3,Fig. 2).

**Table 2.** Trajectory model parameters for all regions and both absolute and proportional volume.

Region	Absolute					Proportional				
		Beta	SE	η *_p_^2^*	Peak age (quadratic models; years)		Beta	SE	η *_p_^2^*	Peak age (quadratic models; years)
Total Gray Matter	Intercept	583988.93	17165.57		6.85	Intercept	56.4318	0.9058		3.67
	Age	46814.57	6410.1	0.16		Age	0.6814	0.3629	0.0110	
	Age ^2^	-3416.89	618.51	0.1		Age ^2^	-0.0927	0.0353	0.0219	
Right Accumbens Area	Intercept	358	33.92		6.06	Intercept	0.0361	0.0027		4.87
	Age	61.5	13.48	0.06		Age	0.0026	0.0011	0.0179	
	Age ^2^	-5.07	1.31	0.05		Age ^2^	-0.0003	0.0001	0.0205	
Left Accumbens Area	Intercept	335.94	33.87		6.05	Intercept	0.0341	0.0026		5.10
	Age	72.26	13.47	0.08		Age	0.0035	0.0011	0.0322	
	Age ^2^	-5.97	1.31	0.06		Age ^2^	-0.0003	0.0001	0.0335	
Right Amygdala	Intercept	580.46	44.25		6.96	Intercept	0.0581	0.0033		5.87
	Age	86.91	17.48	0.07		Age	0.0033	0.0013	0.0179	
	Age ^2^	-6.24	1.69	0.04		Age ^2^	-0.0003	0.0001	0.0141	
Left Amygdala	Intercept	559.39	43.69		6.78	Intercept	0.0576	0.0033		5.74
	Age	102.08	17.33	0.1		Age	0.0041	0.0013	0.0282	
	Age ^2^	-7.53	1.68	0.06		Age ^2^	-0.0004	0.0001	0.0233	
Right Caudate	Intercept	3335.45	98.08		6.69	Intercept	0.3322	0.0034		
	Age	233.03	36.13	0.13		Age	-0.0025	0.0004	0.1161	
	Age ^2^	-17.41	3.49	0.08						
Left Caudate	Intercept	3204.43	91.89		6.91	Intercept	0.3138	0.0068		3.49
	Age	263.16	33.01	0.19		Age	0.0032	0.0025	0.0060	
	Age ^2^	-19.05	3.18	0.11		Age ^2^	-0.0005	0.0002	0.0131	
Right Hippocampus	Intercept	2625.68	112.24		6.85	Intercept	0.2639	0.0085		5.53
	Age	352.11	43.39	0.19		Age	0.0112	0.0033	0.0367	
	Age ^2^	-25.71	4.19	0.12		Age ^2^	-0.001	0.0003	0.0327	
Left Hippocampus	Intercept	2500.39	99.52		6.94	Intercept	0.2516	0.0087		5.64
	Age	335.59	38.29	0.21		Age	0.0107	0.0034	0.0321	
	Age ^2^	-24.18	3.7	0.13		Age ^2^	-0.0009	0.0003	0.0275	
Right Pallidum	Intercept	980.37	17.46			Intercept	0.0881	0.0014		
	Age	34	2.91	0.31		Age	0.0006	0.0002	0.0199	
Left Pallidum	Intercept	1084.66	17.68			Intercept	0.1074	0.0039		5.00
	Age	28.83	2.94	0.24		Age	-0.0043	0.0016	0.0241	
						Age ^2^	0.0004	0.0002	0.0260	
Right Putamen	Intercept	3653.71	165.89		6.3	Intercept	0.3659	0.0124		4.87
	Age	520.28	63.39	0.19		Age	0.0184	0.0048	0.0481	
	Age ^2^	-41.31	6.12	0.14		Age ^2^	-0.0019	0.0005	0.0546	
Left Putamen	Intercept	3469.78	151.3		6.32	Intercept	0.353	0.0112		5.11
	Age	592.87	56.72	0.28		Age	0.0238	0.0043	0.0991	
	Age ^2^	-46.87	5.47	0.21		Age ^2^	-0.0023	0.0004	0.1022	
Right Thalamus Proper	Intercept	5734.55	179.86		7.65	Intercept	0.5673	0.0131		6.05
	Age	575.42	68.29	0.2		Age	0.0126	0.0050	0.0210	
	Age ^2^	-37.59	6.59	0.1		Age ^2^	-0.001	0.0005	0.0156	
Left Thalamus Proper	Intercept	5764.39	170.82		7.55	Intercept	0.5729	0.0131		6.07
	Age	638.25	62.04	0.27		Age	0.0166	0.0049	0.0387	
	Age ^2^	-42.28	5.98	0.15		Age ^2^	-0.0014	0.0005	0.0287	
Right Ventral Diencephalon	Intercept	3060.63	85.44		>8.04	Intercept	0.3002	0.0030		
	Age	273.98	32.17	0.2		Age	0.0054	0.0005	0.3219	
	Age ^2^	-11.14	3.1	0.04						
Left Ventral Diencephalon	Intercept	3266.05	89.36		>8.04	Intercept	0.3221	0.0078		<1.97
	Age	230.2	33.63	0.14		Age	-0.0003	0.0030	0.0000	
	Age ^2^	-5.67	3.25	0.01		Age ^2^	0.0006	0.0003	0.0154	
Left Basal Forebrain	Intercept	628.67	50.09		5.43	Intercept	0.0685	0.0012		
	Age	66.12	19.91	0.03		Age	-0.0011	0.0002	0.0576	
	Age ^2^	-6.09	1.93	0.03						
Right Basal Forebrain	Intercept	765.41	9			Intercept	0.066	0.0010		
						Age	-0.0011	0.0002	0.0822	
Right Anterior Cingulate Gyrus	Intercept	4931.16	341.7		7.96	Intercept	0.482	0.0058		
	Age	354.67	132.88	0.02						
	Age ^2^	-22.29	12.84	0.01						
Left Anterior Cingulate Gyrus	Intercept	5147.96	252.22		6.35	Intercept	0.509	0.0186		3.81
	Age	518.51	92.16	0.1		Age	0.0096	0.0071	0.0064	
	Age ^2^	-40.84	8.89	0.07		Age ^2^	-0.0013	0.0007	0.0119	
Right Anterior Insula	Intercept	2510.23	167.12		7.29	Intercept	0.256	0.0138		6.67
	Age	628.01	61.96	0.27		Age	0.0326	0.0054	0.1088	
	Age ^2^	-43.09	5.98	0.16		Age ^2^	-0.0024	0.0005	0.0692	
Left Anterior Insula	Intercept	2631.93	194.24		7.22	Intercept	0.2721	0.0151		6.59
	Age	657.35	73.4	0.22		Age	0.0329	0.0060	0.0909	
	Age ^2^	-45.55	7.08	0.13		Age ^2^	-0.0025	0.0006	0.0588	
Right Anterior Orbital Gyrus	Intercept	2185.32	183.12		5.96	Intercept	0.2201	0.0048		
	Age	161.26	72.36	0.02		Age	-0.0025	0.0009	0.0255	
	Age ^2^	-13.53	7.01	0.01						
Left Anterior Orbital Gyrus	Intercept	2106.95	53.97			Intercept	0.1851	0.0038		
	Age	29.58	9.15	0.03		Age	-0.0015	0.0007	0.0145	
Right Angular Gyrus	Intercept	11328.55	583.49		6.31	Intercept	1.1164	0.0422		4.51
	Age	1318.64	226.38	0.1		Age	0.0385	0.0168	0.0164	
	Age ^2^	-104.48	21.88	0.07		Age ^2^	-0.0043	0.0016	0.0217	
Left Angular Gyrus	Intercept	9722.66	647.24		6.6	Intercept	0.9501	0.0492		4.86
	Age	1132.58	254.96	0.06		Age	0.0373	0.0196	0.0112	
	Age ^2^	-85.81	24.68	0.04		Age ^2^	-0.0038	0.0019	0.0128	
Right Calcarine Cortex	Intercept	1782.4	405.32		5.39	Intercept	0.1924	0.0322		4.83
	Age	808.09	160.25	0.08		Age	0.0488	0.0128	0.0447	
	Age ^2^	-74.92	15.52	0.07		Age ^2^	-0.005	0.0012	0.0516	
Left Calcarine Cortex	Intercept	3130.63	350.97		5.7	Intercept	0.3088	0.0284		4.43
	Age	483.7	137.65	0.04		Age	0.0204	0.0112	0.0109	
	Age ^2^	-42.44	13.32	0.03		Age ^2^	-0.0023	0.0011	0.0150	
Right Central Operculum	Intercept	4440.69	79.56			Intercept	0.4218	0.0144		6.72
	Age	46.86	11.87	0.05		Age	-0.0175	0.0057	0.0304	
						Age ^2^	0.0013	0.0005	0.0185	
Left Central Operculum	Intercept	4429.78	83.91			Intercept	0.4244	0.0134		5.91
	Age	75.69	11.66	0.13		Age	-0.0166	0.0052	0.0325	
						Age ^2^	0.0014	0.0005	0.0254	
Right Cuneus	Intercept	5537.99	295.43		4.75	Intercept	0.5409	0.0084		
	Age	254.4	113.96	0.02		Age	-0.012	0.0013	0.2171	
	Age ^2^	-26.8	11.01	0.02						
Left Cuneus	Intercept	5579.91	299.5		4.3	Intercept	0.5385	0.0084		
	Age	203.05	115.81	0.01		Age	-0.0135	0.0014	0.2520	
	Age ^2^	-23.6	11.19	0.02						
Right Entorhinal Area	Intercept	812.61	142.81		6.39	Intercept	0.0916	0.0113		5.76
	Age	325.16	57.35	0.09		Age	0.0176	0.0046	0.0399	
	Age ^2^	-25.45	5.58	0.06		Age ^2^	-0.0015	0.0004	0.0326	
Left Entorhinal Area	Intercept	929.07	128.94		6.37	Intercept	0.0969	0.0100		5.67
	Age	278.11	51.65	0.08		Age	0.0152	0.0040	0.0400	
	Age ^2^	-21.82	5.02	0.06		Age ^2^	-0.0013	0.0004	0.0338	
Right Frontal Operculum	Intercept	1947.16	129.9		>8.04	Intercept	0.1963	0.0026		
	Age	163.03	49.97	0.04						
	Age ^2^	-10.13	4.83	0.02						
Left Frontal Operculum	Intercept	2435.27	55.49			Intercept	0.2154	0.0028		
	Age	57.22	8.68	0.13						
Right Frontal Pole	Intercept	3245.01	290.38		6.97	Intercept	0.3182	0.0220		5.77
	Age	495.29	113.19	0.06		Age	0.0214	0.0087	0.0195	
	Age ^2^	-35.56	10.94	0.03		Age ^2^	-0.0019	0.0008	0.0159	
Left Frontal Pole	Intercept	2618.07	307.6		6.06	Intercept	0.2667	0.0232		5.13
	Age	611.18	120.6	0.08		Age	0.0306	0.0092	0.0332	
	Age ^2^	-50.46	11.67	0.06		Age ^2^	-0.003	0.0009	0.0341	
Right Fusiform Gyrus	Intercept	5417.05	361.49		7.05	Intercept	0.5444	0.0266		5.99
	Age	920.61	139.59	0.13		Age	0.0388	0.0105	0.0420	
	Age ^2^	-65.28	13.49	0.08		Age ^2^	-0.0032	0.0010	0.0321	
Left Fusiform Gyrus	Intercept	5588.66	319.21		6.67	Intercept	0.5688	0.0240		5.62
	Age	1020.33	122.37	0.2		Age	0.0434	0.0094	0.0664	
	Age ^2^	-76.52	11.82	0.13		Age ^2^	-0.0039	0.0009	0.0576	
Right Gyrus Rectus	Intercept	1927.64	79.66			Intercept	0.1722	0.0059		
	Age	76.1	14.7	0.07		Age	0.0019	0.0011	0.0077	
									
Left Gyrus Rectus	Intercept	1881.86	70.22			Intercept	0.1673	0.0052		
	Age	74.57	13.11	0.09		Age	0.0021	0.0010	0.0119	
									
Right Inferior Occipital Gyrus	Intercept	6330.21	533.2		5.66	Intercept	0.6222	0.0409		3.97
	Age	801.36	209.86	0.05		Age	0.0268	0.0164	0.0082	
	Age ^2^	-70.73	20.31	0.04		Age ^2^	-0.0034	0.0016	0.0141	
Left Inferior Occipital Gyrus	Intercept	6982.51	541.4		5.66	Intercept	0.7319	0.0146		
	Age	634.76	211.82	0.03		Age	-0.0102	0.0027	0.0427	
	Age ^2^	-56.09	20.49	0.02						
Right Inferior Temporal Gyrus	Intercept	9254.63	647.56		6.95	Intercept	0.9933	0.0076		
	Age	1087.08	255.43	0.06						
	Age ^2^	-78.24	24.73	0.03						
Left Inferior Temporal Gyrus	Intercept	8836.32	541.29		7.29	Intercept	0.8646	0.0403		5.95
	Age	1100.77	211.71	0.08		Age	0.0389	0.0161	0.0174	
	Age ^2^	-75.51	20.48	0.04		Age ^2^	-0.0033	0.0016	0.0133	
Right Lingual Gyrus	Intercept	8406.36	585.84		5.93	Intercept	0.8299	0.0435		3.88
	Age	934.45	230.45	0.05		Age	0.0232	0.0174	0.0055	
	Age ^2^	-78.85	22.3	0.04		Age ^2^	-0.003	0.0017	0.0099	
Left Lingual Gyrus	Intercept	8124.78	491.89		6.76	Intercept	0.8238	0.0122		
	Age	669.04	193.63	0.04		Age	-0.0045	0.0022	0.0125	
	Age ^2^	-49.48	18.74	0.02						
Right Lateral Orbital Gyrus	Intercept	1935.9	216.17		6.56	Intercept	0.2112	0.0031		
	Age	250.86	85.61	0.03						
	Age ^2^	-19.13	8.3	0.02						
Left Lateral Orbital Gyrus	Intercept	2362.1	222.51		5.66	Intercept	0.2454	0.0058		
	Age	203.03	87.43	0.02		Age	-0.0035	0.0010	0.0360	
	Age ^2^	-17.95	8.46	0.02						
Right Middle Cingulate Gyrus	Intercept	4847.72	261.32		6.69	Intercept	0.4614	0.0068		
	Age	218.98	102.17	0.02		Age	-0.0054	0.0012	0.0595	
	Age ^2^	-16.36	9.88	0.01						
Left Middle Cingulate Gyrus	Intercept	5728.89	82.73			Intercept	0.5075	0.0092		
						Age	-0.0112	0.0017	0.1244	
Right Medial Frontal Cortex	Intercept	1745	151.46		6.67	Intercept	0.1966	0.0023		
	Age	249.28	59.23	0.06						
	Age ^2^	-18.69	5.73	0.03						
Left Medial Frontal Cortex	Intercept	1817.71	130.23		6.66	Intercept	0.186	0.0024		
	Age	179.1	50.37	0.04						
	Age ^2^	-13.45	4.87	0.03						
Right Middle Frontal Gyrus	Intercept	20659.61	957.37		6.95	Intercept	2.0252	0.0645		5.45
	Age	2448.85	365.87	0.14		Age	0.0776	0.0256	0.0292	
	Age ^2^	-176.11	35.33	0.08		Age ^2^	-0.0071	0.0025	0.0267	
Left Middle Frontal Gyrus	Intercept	21464.61	1165.26		7.19	Intercept	2.189	0.0129		
	Age	2012.08	453.42	0.06						
	Age ^2^	-139.88	43.83	0.03						
Right Middle Occipital Gyrus	Intercept	6305.45	347.16		5.16	Intercept	0.605	0.0274		<1.97
	Age	443	134.55	0.04		Age	0.0035	0.0109	0.0003	
	Age ^2^	-42.92	13	0.04		Age ^2^	-0.0015	0.0011	0.0069	
Left Middle Occipital Gyrus	Intercept	6378.42	375.13		5.45	Intercept	0.6328	0.0103		
	Age	377.32	146.28	0.02		Age	-0.0106	0.0018	0.1049	
	Age ^2^	-34.62	14.14	0.02						
Right Medial Orbital Gyrus	Intercept	3023.58	314.21		7.42	Intercept	0.3095	0.0033		
	Age	285.43	125.29	0.02						
	Age ^2^	-19.22	12.16	0.01						
Left Medial Orbital Gyrus	Intercept	2822.34	317.43		6.44	Intercept	0.3126	0.0033		
	Age	392.34	126.82	0.03						
	Age ^2^	-30.44	12.31	0.02						
Right Postcentral Gyrus Medial Segment	Intercept	1827.15	154.01		6.48	Intercept	0.1675	0.0120		6.78
	Age	-189.29	61.26	0.03		Age	-0.0222	0.0048	0.0640	
	Age ^2^	14.62	5.94	0.02		Age ^2^	0.0016	0.0005	0.0388	
Left Postcentral Gyrus Medial Segment	Intercept	1624.66	149.71		6.24	Intercept	0.1538	0.0116		6.66
	Age	-148.95	59.59	0.02		Age	-0.0196	0.0046	0.0554	
	Age ^2^	11.93	5.78	0.01		Age ^2^	0.0015	0.0004	0.0346	
Right Precentral Gyrus Medial Segment	Intercept	2964.09	46.22			Intercept	0.3069	0.0144		6.78
						Age	-0.0242	0.0057	0.0546	
						Age ^2^	0.0018	0.0006	0.0330	
Left Precentral Gyrus Medial Segment	Intercept	3530.48	180		5.32	Intercept	0.3296	0.0129		6.46
	Age	-167.38	71.27	0.02		Age	-0.0284	0.0051	0.0899	
	Age ^2^	15.73	6.91	0.02		Age ^2^	0.0022	0.0005	0.0604	
Right Superior Frontal Gyrus Medial Segment	Intercept	8043.09	426.09		7.24	Intercept	0.7768	0.0111		
	Age	487.94	164.32	0.03		Age	-0.0043	0.0018	0.0175	
	Age ^2^	-33.69	15.87	0.02						
Left Superior Frontal Gyrus Medial Segment	Intercept	6643.02	346.98		7.01	Intercept	0.6832	0.0055		
	Age	651.14	133.26	0.08						
	Age ^2^	-46.45	12.87	0.04						
Right Middle Temporal Gyrus	Intercept	12247.91	645.07		6.68	Intercept	1.2415	0.0493		5.62
	Age	2223.14	248.79	0.22		Age	0.0963	0.0196	0.0726	
	Age ^2^	-166.52	24.03	0.14		Age ^2^	-0.0086	0.0019	0.0629	
Left Middle Temporal Gyrus	Intercept	12876.2	636.54		6.67	Intercept	1.2952	0.0466		5.36
	Age	1858.37	246.47	0.16		Age	0.0646	0.0185	0.0374	
	Age ^2^	-139.24	23.82	0.11		Age ^2^	-0.006	0.0018	0.0354	
Right Occipital Pole	Intercept	2160.26	134.57			Intercept	0.1871	0.0051		
	Age	44.09	24.15	0.01						
Left Occipital Pole	Intercept	1826.7	158			Intercept	0.1668	0.0122		
	Age	144.78	28.23	0.08		Age	0.0065	0.0022	0.0248	
Right Occipital Fusiform Gyrus	Intercept	5511.29	73.3			Intercept	0.4891	0.0086		
						Age	-0.011	0.0016	0.1209	
									
Left Occipital Fusiform Gyrus	Intercept	5546.75	123.19			Intercept	0.4836	0.0087		
	Age	-33.24	20.84	0.01		Age	-0.0117	0.0016	0.1503	
Right Opercular Part of the Inferior Frontal Gyrus	Intercept	3681.42	229.38		6.71	Intercept	0.3765	0.0045		
	Age	357.71	88.7	0.05						
	Age ^2^	-26.67	8.57	0.03						
Left Opercular Part of the Inferior Frontal Gyrus	Intercept	3322.39	245.61		6.53	Intercept	0.3361	0.0050		
	Age	315.87	94.42	0.04						
	Age ^2^	-24.19	9.12	0.02						
Right Orbital Part of the Inferior Frontal Gyrus	Intercept	1532.06	164.86		7.03	Intercept	0.1521	0.0129		5.89
	Age	228.8	64.34	0.04		Age	0.0089	0.0051	0.0099	
	Age ^2^	-16.27	6.22	0.02		Age ^2^	-0.0008	0.0005	0.0077	
Left Orbital Part of the Inferior Frontal Gyrus	Intercept	1787.83	172.67		7.7	Intercept	0.1814	0.0028		
	Age	158.03	67.65	0.02						
	Age ^2^	-10.26	6.54	0.01						
Right Posterior Cingulate Gyrus	Intercept	3805.79	243.48		7.09	Intercept	0.4033	0.0033		
	Age	420.84	94.94	0.06						
	Age ^2^	-29.69	9.18	0.03						
Left Posterior Cingulate Gyrus	Intercept	5097.95	227.01		6.67	Intercept	0.4967	0.0060		
	Age	307.02	86.02	0.04		Age	-0.0045	0.0010	0.0700	
	Age ^2^	-23.02	8.3	0.03						
Right Precuneus	Intercept	11495.34	553.6		6.64	Intercept	1.1153	0.0385		3.50
	Age	956.01	214.3	0.06		Age	0.0148	0.0152	0.0032	
	Age ^2^	-71.99	20.71	0.04		Age ^2^	-0.0021	0.0015	0.0070	
Left Precuneus	Intercept	12471.82	514.69		6.04	Intercept	1.2267	0.0132		
	Age	754.89	196.56	0.05		Age	-0.0153	0.0022	0.1423	
	Age ^2^	-62.5	18.98	0.04						
Right Parahippocampal Gyrus	Intercept	2268.76	210.14		6.88	Intercept	0.2434	0.0026		
	Age	264.82	83.76	0.03						
	Age ^2^	-19.25	8.13	0.02						
Left Parahippocampal Gyrus	Intercept	2354.36	236.55		6.33	Intercept	0.2396	0.0186		5.11
	Age	391.49	94.22	0.05		Age	0.015	0.0075	0.0122	
	Age ^2^	-30.91	9.14	0.04		Age ^2^	-0.0015	0.0007	0.0126	
Right Posterior Insula	Intercept	1267.39	165.7		7.48	Intercept	0.1295	0.0134		6.86
	Age	352.12	63.6	0.1		Age	0.0191	0.0053	0.0425	
	Age ^2^	-23.55	6.14	0.05		Age ^2^	-0.0014	0.0005	0.0249	
Left Posterior Insula	Intercept	1784.04	109.07		6.71	Intercept	0.1824	0.0087		5.68
	Age	316.03	41.51	0.17		Age	0.0125	0.0034	0.0419	
	Age ^2^	-23.56	4.01	0.11		Age ^2^	-0.0011	0.0003	0.0355	
Right Parietal Operculum	Intercept	2235.13	152.59		7.41	Intercept	0.2148	0.0029		
	Age	152.28	58.84	0.02						
	Age ^2^	-10.27	5.68	0.01						
Left Parietal Operculum	Intercept	2733.54	66.07			Intercept	0.2656	0.0118		4.87
	Age	72.58	8.94	0.19		Age	-0.0093	0.0045	0.0146	
						Age ^2^	0.001	0.0004	0.0166	
Right Postcentral Gyrus	Intercept	14625.84	825.75		5.78	Intercept	1.3409	0.0595		6.84
	Age	-639.36	329	0.01		Age	-0.1055	0.0239	0.0551	
	Age ^2^	55.33	31.92	0.01		Age ^2^	0.0077	0.0023	0.0327	
Left Postcentral Gyrus	Intercept	14695.28	158.92			Intercept	1.3941	0.0717		8.22
						Age	-0.0688	0.0289	0.0165	
						Age ^2^	0.0042	0.0028	0.0066	
Right Posterior Orbital Gyrus	Intercept	2560.29	167.62		6.25	Intercept	0.2526	0.0129		4.32
	Age	292.09	64.52	0.07		Age	0.0079	0.0051	0.0079	
	Age ^2^	-23.37	6.23	0.05		Age ^2^	-0.0009	0.0005	0.0114	
Left Posterior Orbital Gyrus	Intercept	2743.39	183.34		6.61	Intercept	0.2679	0.0135		4.75
	Age	302.13	70.3	0.06		Age	0.0089	0.0053	0.0095	
	Age ^2^	-22.85	6.79	0.04		Age ^2^	-0.0009	0.0005	0.0114	
Right Planum Polare	Intercept	1610.37	130.92		6.33	Intercept	0.1574	0.0104		5.08
	Age	245.58	51.75	0.07		Age	0.0109	0.0042	0.0207	
	Age ^2^	-19.4	5.01	0.05		Age ^2^	-0.0011	0.0004	0.0217	
Left Planum Polare	Intercept	1947.47	115.61		6.72	Intercept	0.1984	0.0019		
	Age	186.66	44.85	0.06						
	Age ^2^	-13.88	4.33	0.03						
Right Precentral Gyrus	Intercept	14026.16	243.9			Intercept	1.2297	0.0151		
	Age	98.69	39.95	0.02		Age	-0.0168	0.0028	0.1000	
Left Precentral Gyrus	Intercept	13601.13	249.18			Intercept	1.1952	0.0161		
	Age	171.41	42.17	0.05		Age	-0.0108	0.0029	0.0400	
Right Planum Temporale	Intercept	1912.83	144.18		6.94	Intercept	0.1875	0.0040		
	Age	117.65	56.35	0.01		Age	-0.0016	0.0007	0.0183	
	Age ^2^	-8.48	5.45	0.01						
Left Planum Temporale	Intercept	2166.01	151.19		6.83	Intercept	0.214	0.0042		
	Age	146.28	58.35	0.02		Age	-0.0017	0.0007	0.0208	
	Age ^2^	-10.72	5.64	0.01						
Right Subcallosal Area	Intercept	1042.87	102.15		6.04	Intercept	0.1009	0.0078		4.16
	Age	118.28	39.85	0.03		Age	0.0038	0.0031	0.0048	
	Age ^2^	-9.8	3.85	0.02		Age ^2^	-0.0005	0.0003	0.0075	
Left Subcallosal Area	Intercept	954.01	110.34		6.11	Intercept	0.0993	0.0016		
	Age	110.44	43.3	0.02						
	Age ^2^	-9.03	4.19	0.02						
Right Superior Frontal Gyrus	Intercept	15938.7	361.89			Intercept	1.3853	0.0100		
	Age	324.64	63.26	0.08						
Left Superior Frontal Gyrus	Intercept	16159.9	382.35			Intercept	1.4107	0.0113		
	Age	341.05	68.4	0.07						
Right Supplementary Motor Cortex	Intercept	5383.12	121.11			Intercept	0.5214	0.0241		6.29
	Age	62.8	20.98	0.03		Age	-0.0249	0.0096	0.0200	
						Age ^2^	0.002	0.0009	0.0138	
Left Supplementary Motor Cortex	Intercept	5079.55	128.69			Intercept	0.4497	0.0049		
	Age	123.85	22.08	0.09						
Right Supramarginal Gyrus	Intercept	7633.08	566.58		6.03	Intercept	0.7723	0.0430		4.79
	Age	1302.41	221.57	0.1		Age	0.0533	0.0169	0.0321	
	Age ^2^	-107.98	21.43	0.08		Age ^2^	-0.0056	0.0016	0.0376	
Left Supramarginal Gyrus	Intercept	8416.9	701.74		6.36	Intercept	0.874	0.0093		
	Age	910.31	278.09	0.03						
	Age ^2^	-71.6	26.95	0.02						
Right Superior Occipital Gyrus	Intercept	4056.62	376.4		5.83	Intercept	0.4011	0.0296		4.07
	Age	510.03	149.87	0.04		Age	0.0168	0.0119	0.0062	
	Age ^2^	-43.74	14.54	0.03		Age ^2^	-0.0021	0.0012	0.0101	
Left Superior Occipital Gyrus	Intercept	4868.44	130.05			Intercept	0.4274	0.0097		
	Age	31.75	22.26	0.01		Age	-0.006	0.0017	0.0362	
Right Superior Parietal Lobule	Intercept	13215.42	183.88			Intercept	1.3111	0.0793		7.78
						Age	-0.0826	0.0320	0.0186	
						Age ^2^	0.0053	0.0031	0.0083	
Left Superior Parietal Lobule	Intercept	13399.46	183.2			Intercept	1.1633	0.0234		
						Age	-0.0214	0.0044	0.0647	
Right Superior Temporal Gyrus	Intercept	7614.21	422.26		6.35	Intercept	0.7407	0.0309		3.66
	Age	696.26	163.43	0.06		Age	0.0142	0.0122	0.0045	
	Age ^2^	-54.83	15.79	0.04		Age ^2^	-0.0019	0.0012	0.0091	
Left Superior Temporal Gyrus	Intercept	6565.77	467.44		6.4	Intercept	0.6424	0.0350		5.13
	Age	982.46	183.29	0.09		Age	0.0425	0.0140	0.0281	
	Age ^2^	-76.78	17.73	0.06		Age ^2^	-0.0041	0.0014	0.0289	
Right Temporal Pole	Intercept	4629.59	389.27		7.09	Intercept	0.4802	0.0302		6.34
	Age	1220.26	152.34	0.18		Age	0.0642	0.0120	0.0853	
	Age ^2^	-86.04	14.74	0.1		Age ^2^	-0.0051	0.0012	0.0592	
Left Temporal Pole	Intercept	4623.86	448.04		7.24	Intercept	0.4772	0.0336		6.57
	Age	1122.6	176.04	0.12		Age	0.0561	0.0134	0.0526	
	Age ^2^	-77.53	17.03	0.07		Age ^2^	-0.0043	0.0013	0.0337	
Right Triangular Part of the Inferior Frontal Gyrus	Intercept	2615.03	312.09		5.84	Intercept	0.2841	0.0236		5.17
	Age	888.85	123.2	0.15		Age	0.0467	0.0094	0.0732	
	Age ^2^	-76.07	11.93	0.12		Age ^2^	-0.0045	0.0009	0.0744	
Left Triangular Part of the Inferior Frontal Gyrus	Intercept	4225.5	314.48		>8.04	Intercept	0.4177	0.0085		
	Age	342.3	123.8	0.02		Age	0.0021	0.0014	0.0066	
	Age ^2^	-19.48	11.98	0.01						
Right Transverse Temporal Gyrus	Intercept	1780.69	33.83			Intercept	0.174	0.0096		7.81
						Age	-0.0103	0.0038	0.0241	
						Age ^2^	0.0007	0.0004	0.0109	
Left Transverse Temporal Gyrus	Intercept	1893.66	50.44			Intercept	0.1815	0.0078		6.27
	Age	24.73	6.17	0.05		Age	-0.0078	0.0029	0.0245	
						Age ^2^	0.0006	0.0003	0.0170	

For some regions, predicted age at peak volume was outside of the observed age range and peak age is displayed at >8.04 and <1.97, respectively.

### 3.2 Regional development trajectories for absolute volume (mm ^3^ )

The best fitting model (i.e., the lowest AIC) was quadratic for 88 of 116 regions (76%), linear for 20 regions (17%), and null for 8 regions (7%); seeTables 2,S1. In regions where the null trajectory was the best fit, null models were about twice as likely to be the best fitting model than the next-best fitting age trajectory model.

**Fig. 3.**
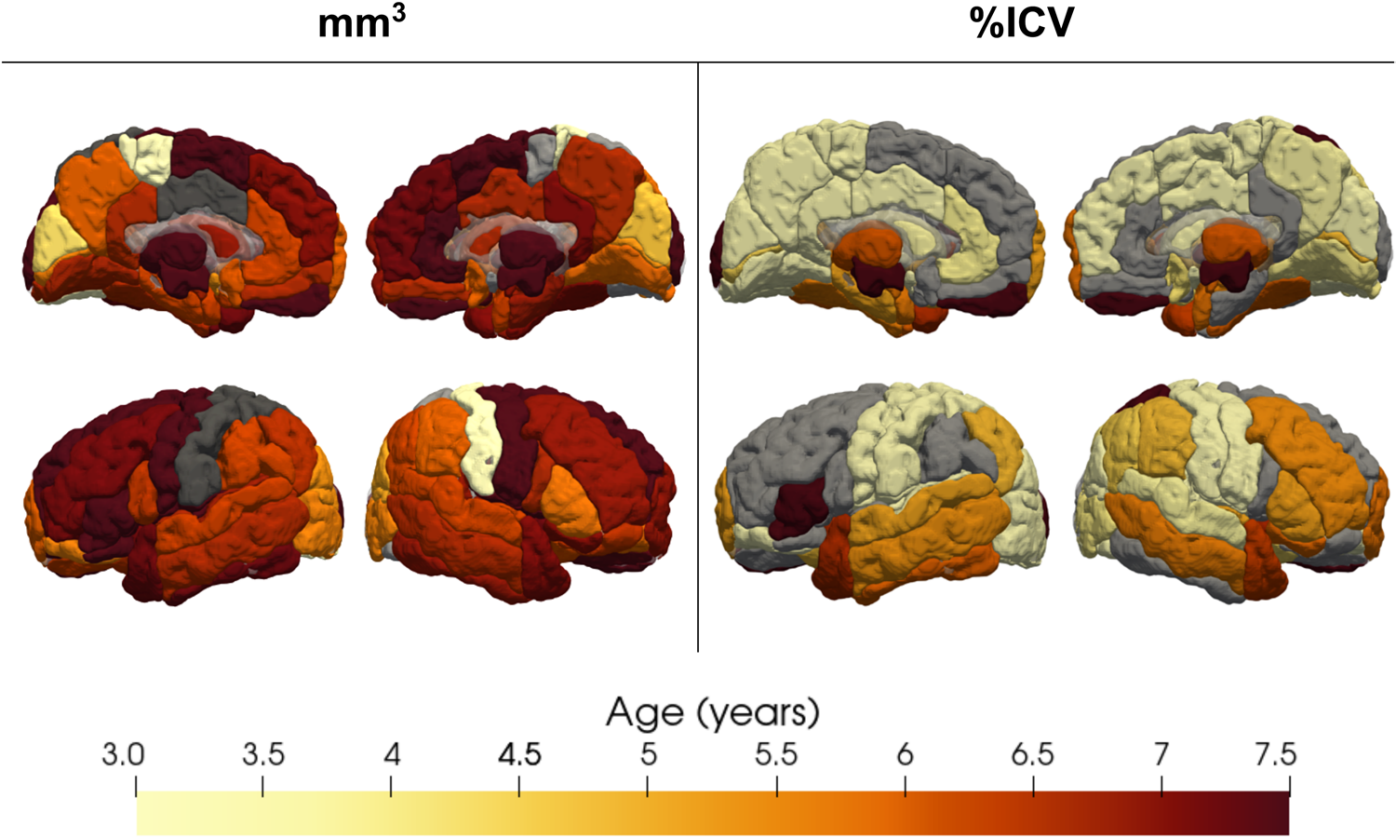
Age at peak absolute volume (left), and proportional volume (right). For most regions, absolute volume reaches a peak around age 6 or later, consistent with our finding that absolute volumes primarily increase between ages 2 and 8 years. On the other hand, proportional volume was largest for most regions before age 5.5, consistent with the finding that proportional volume shows a net decrease between ages 2 and 8 years. For regions with a linearly decreasing trajectory, peak age is coded as 3 years and linearly increasing trajectories are coded as 7.5 years. Regions with a null trajectory are shown in gray.

**Fig. 4.**
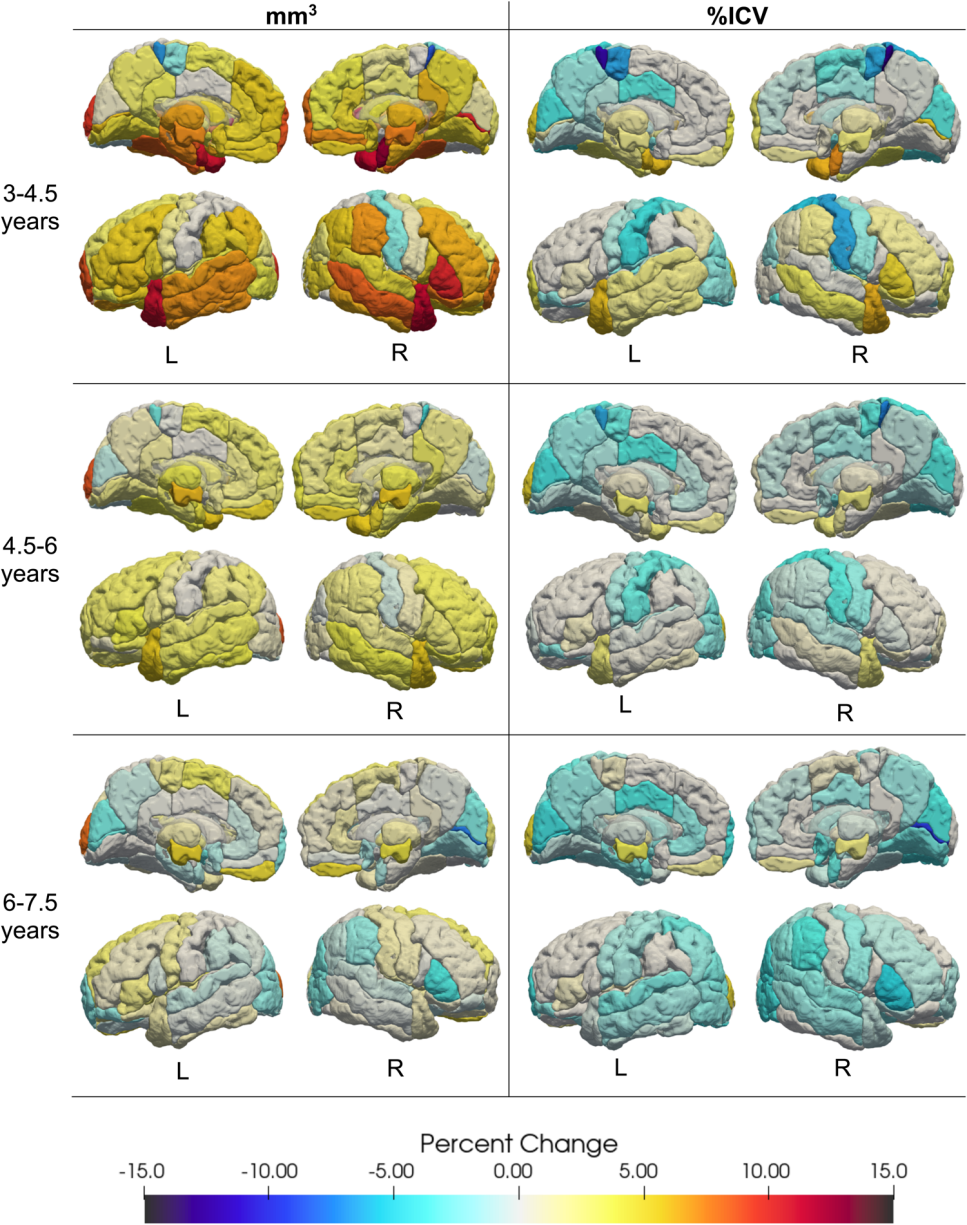
Percent change for absolute regional volume (left) and proportional volume normalized as %ICV (right). Most absolute volumes increased between 2 and 8 years, while proportional volume generally decreased. Development followed a rostro-caudal pattern with posterior regions decreasing in volume earlier than frontal regions. Regions with a null development trajectory are coded as a 0% change.

We generally observed earlier peaks in posterior regions and later peaks in anterior regions (Fig. 3;Table 2). The age at peak volume for quadratic trajectories ranged from 4.03 (left cuneus) to 7.96 years (right anterior cingulate gyrus). Most regions showed larger volumes at 8 years than 2 years, though rates of development differed (Fig. 4). Regions with large changes in volume included the temporal pole (right: 23%,*η_p_^2^_age_*= 0.18; left: 23.3%,*η_p_^2^_age_*= 0.12) and gyrus rectus (right: 21.56%,*η_p_^2^_age_*= 0.07; left: 21.63%,*η_p_^2^_age_*= 0.09). Three regions showed highly asymmetric changes: the occipital pole (right: 11.65%,*η_p_^2^_age_*= 0.01; left: 39.69%,*η_p_^2^_age_*= 0.08), anterior insula (right: 24%,*η_p_^2^_age_*= 0.27, left: 4%,*η_p_^2^_age_*= 0.22), and posterior insula (right: 27.93%,*η_p_^2^_age_*= 0.10; left: 14.07%,*η_p_^2^_age_*= 0.17). Additional regions with strong age effects included the right and left pallidum (right: 19.15%,*η_p_^2^_age_*= 0.31; left: 14.96%,*η_p_^2^_age_*= 0.24), left hippocampus (13.10%,*η_p_^2^_age_*= 0.21), left putamen (9.96%,*η_p_^2^_age_*= 0.21,*η_p_^2^_age2_*= 0.28), left thalamus (14.64%*η_p_^2^_age_*= 0.27), right middle temporal gyrus (14.01%,*η_p_^2^_age_*= 0.21), and right ventral diencephalon (24.71%,*η_p_^2^_age_*= 0.20). Regions that decreased in volume (Fig. 3) included the cuneus (right: -4%,*η_p_^2^_age_*= 0.02; left: -6%,*η_p_^2^_age_*= 0.01,*η_p_^2^_age2_*= 0.02), medial segment of the postcentral gyrus (right: -11%,*η_p_^2^_age_*= 0.03; left: -8%,*η_p_^2^_age_*= 0.02), left occipital fusiform gyrus (-4%,*η_p_^2^_age_*= 0.01), right calcarine cortex (-3%,*η_p_^2^_age_*= 0.08), and right middle occipital gyrus (-2%,*η_p_^2^_age_*= 0.04).

### 3.3 Proportional regional volume development (%ICV)

The best fit for 62 of 116 regions (53%) was the quadratic model, 30 regions (26%) were best fit by a linear model, and 24 regions (21%) were best fit by a null model (Table S2). In regions where the null trajectory was the best fit, null models were about twice as likely to be the best fitting model than the next-best fitting age trajectory model. 54% of regions decreased in proportional volume between 2 and 8 years, while 25% showed an increase (Fig. 4.) The medial postcentral gyrus had the largest proportional decrease (right: -24%,*η_p_^2^_age_*= 0.06; left: -22%,*η_p_^2^_age_*= 0.06), while the left occipital pole had the largest proportional increase (21%,*η_p_^2^_age_*= 0.02). Additional regions with strong age effects included the right ventral diencephalon (10.32% increase,*η_p_^2^_age_*= 0.32), and the cuneus (right: 14.04% decrease,*η_p_^2^_age_*= 0.22, left: 16.07% decrease,*η_p_^2^_age_*= 0.25;Table 2).

#### 3.3.1 Effects of sex

No regions had significant sex interactions for absolute volume. In reduced models (sex interactions removed), 86% of regions had a significant main effect of sex (Table S2); in all regions, males had larger gray matter volume than females. Most sex main effects were medium (*η_p_^2^*> 0.0588*)*to large (*η_p_^2^>*0.1379;Cohen, 2013). In 89 of the regions best fit by a sex main effects model, Akaike weights indicated that a model with sex main effects was at least twice as likely to be the best fitting model than a model with no sex effects. In 66 of those regions, models with a main effect of sex were at least 50 times likelier to be the best fitting model than a model without a sex main effect.

**Fig. 5.**
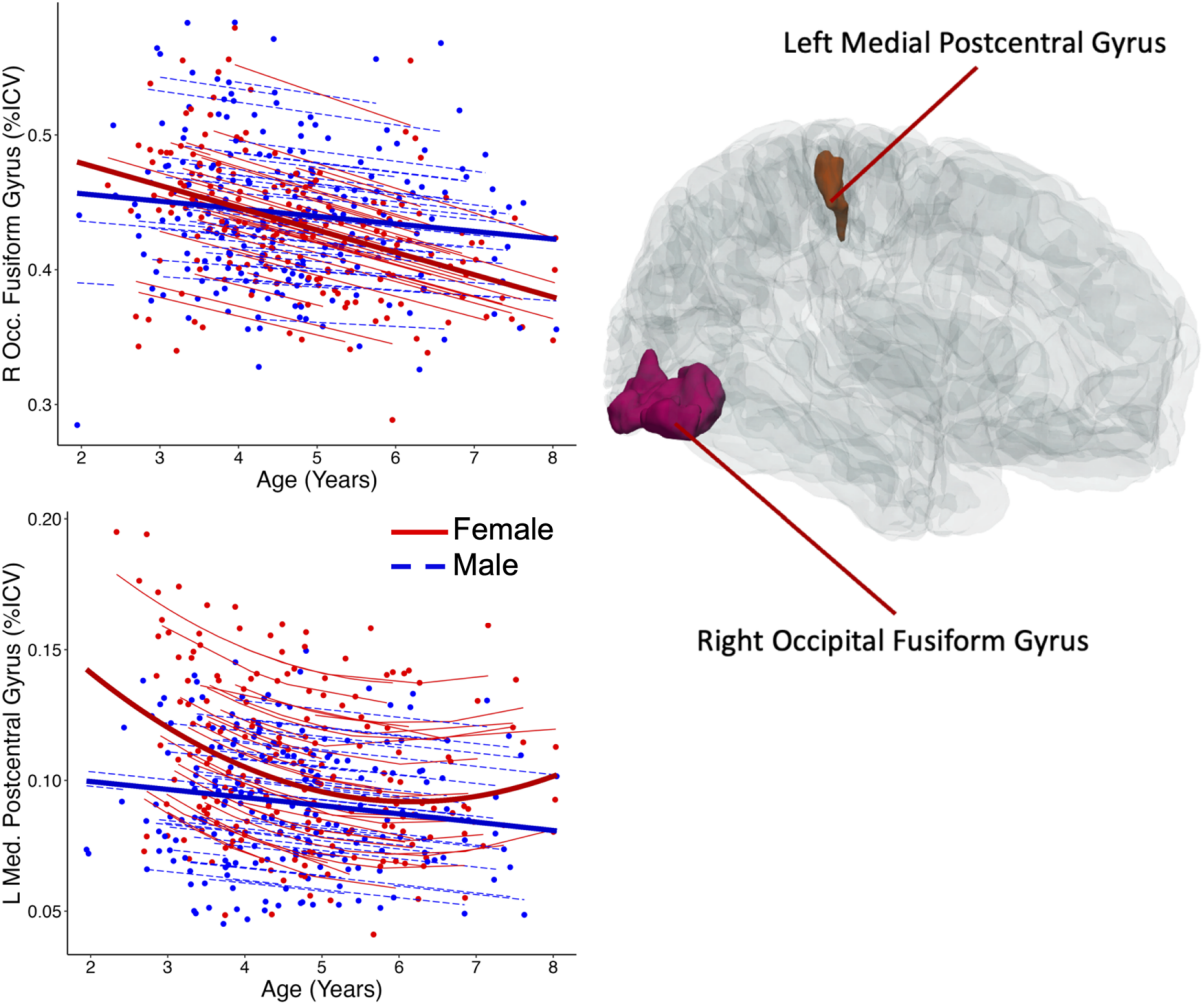
Only two regions had significant sex-by-age effects on proportional volume. In the left medial segment of the postcentral gyrus, females showed a curvilinear decrease while males showed a gradual linear decrease. In the right occipital fusiform gyrus, males showed a more gradual volume decrease than females. Dots represent the proportional volume for a single subject at a single scanning timepoint. Thin lines represent individual subject best fit lines; thick lines represent the group-level trend.

For proportional volume, two regions showed significant sex interactions (development trajectory differences by sex). The left medial postcentral gyrus had a significant main effect of sex (*q*= 0.01,*η_p_^2^*= 0.049) as well as significant sex by age (*q*< 0.04,*η_p_^2^*= 0.039) and sex by age^2^(*q=*0.048,*η_p_^2^*= 0.034) interactions (Fig. 5,Table 3). Females showed a U-shaped trajectory with a rapid decrease from age ~2-6 years while males showed a gradual, linear decrease. The interaction model was 133 times likelier than the main effects only model to be the best fit given the data, and 74 times likelier to be the best fitting model than the model with no sex effects. In the right occipital fusiform gyrus, there was significant sex-by-age interaction (*q*< 0.04,*η_p_^2^*= 0.034;Table 3) with females’ volume decreased more rapidly than males. The interaction model was 225 times likelier to be the best-fitting model than the main effects only model and 1312 times likelier than the model with no sex effects, given the present data. In reduced models, 15% of regions had a main effect of sex on proportional volumes (Table S3). Of those regions, females had larger proportional volume in 10 of them: bilaterally in the caudate, pallidum, and thalamus, the right ventral DC, the left hippocampus, postcentral gyrus, and gyrus rectus. Males had proportionally larger volume in four regions: the left anterior cingulate cortex, inferior occipital gyrus, and middle cingulate gyrus, as well as the right posterior insula. Evidence in favor of a model with main effects of sex over a model without sex effects was particularly strong for the bilateral caudate and pallidum, and the left inferior occipital gyrus and ventral diencephalon, whose sex main effects models were more than 400 times likelier to be the best-fitting model than models with no sex effects.

**Table 3.** Model summaries for regions with significant age-by-sex interactions.

Region as %ICV		Beta	SE	η *_p_^2^*	p	q
Left Postcentral Gyrus Medial Segment	Intercept	0.2001	0.0159			
	Age	-0.0352	0.0063	0.0541		
	Sex	-0.0945	0.0227	0.0490	<.001	0.010 *
	Age ^2^	0.0029	0.0006	0.0333		
	Age * Sex	0.0322	0.0090	0.0393	<.001	0.035 *
	Age ^ 2 *^ Sex	-0.0029	0.0009	0.0341	0.001	0.048 *
Right Occipital Fusiform Gyrus	Intercept	0.5122	0.0123			
	Age	-0.0166	0.0023	0.1261		
	Sex	-0.0446	0.0169	0.0173	0.009	0.183
	Age * Sex	0.0110	0.0032	0.0341	0.001	0.037 *

* indicates that model component was statistically significant after correction for multiple comparisons.

## 4 Discussion

In this study, using longitudinal sampling, we provide a detailed characterization of typical gray matter volume development between 2 and 8 years across 116 gray matter regions. The three key results are: 1) absolute gray matter volume development shows a spatiotemporal pattern where posterior regions typically reach peak volumes earlier than anterior regions; 2) absolute regional volumes generally increase between 2 and 8 years, while proportional volumes are stable or decrease; and 3) there are few sex differences in development trajectories.

### 4.1 Spatiotemporal development pattern

Our finding that posterior regions show absolute volume decreases earlier than anterior regions is broadly consistent with previous literature (Brown & Jernigan, 2012;Lenroot & Giedd, 2006;Lenroot et al., 2007;Tanaka et al., 2012;Uematsu et al., 2012), which used wider age ranges with relatively few children under 8 years who were scanned longitudinally. Thus, our study confirms the presence of an overall posterior-to-anterior developmental pattern in a sample of younger children, complementing what is already known about human neurodevelopment (Ouyang et al., 2019).

In contrast to prior studies however, most regions in our study reached peak volume between 4 and 7 years, with global gray matter volume peaking around 7 years. Previous longitudinal studies showed peak gray matter volumes between ages 9 and 11 years for frontal gray matter, around 8 years for parietal gray matter, and between 10 and 11.5 years for temporal gray matter (Lenroot et al., 2007;Tanaka et al., 2012). Our observations are more similar to prior findings in occipital regions, which peaked around 7 years (Lenroot et al., 2007). A likely reason for differences in peak ages identified between studies are differences in the age range from one sample to another. For example, the oldest child included in our sample was 8.04 years old, whereas Lenroot and colleagues included participants up to 23 years (2007). It is well documented that fitting and interpreting quadratic models for biological data is highly influenced by the age range of the sample (Fjell et al., 2010) and our finding of an earlier peak may be attributable to model fit artifacts of a parabola within the range of ages we sampled. Additionally, quality control procedures for structural neuroimaging data can affect the outcome of the analysis (Ducharme et al., 2016). More recently-available quality control methods may also be more stringent, therefore shifting the shape of the best fitting model towards earlier peaks. When development follows a parabolic trajectory, the slowest development occurs near the peak, which is what we observed in the present study for most regions. While the peaks we found do not precisely concur with findings from previous research including older children and adolescents, our study and previous studies both indicate that developmental changes are relatively slow around ages 7–8 years (Brown & Jernigan, 2012;Lenroot et al., 2007). While the nominal age at peak volume differs between the present study and others, a similar conclusion can be drawn from all of them: developmental changes in gray matter volume occur more slowly during middle to late childhood as absolute gray matter volume approaches a peak, compared to earlier or later childhood.

While a quadratic trajectory was the best fit for most regions for both absolute (mm^3^) and proportional volume (%ICV), roughly one-fifth of regions followed a linear trajectory. A quadratic fit indicates that the rate of change, changes during the course of development from 2–8 years (i.e., sometimes development is fast, other times development is slower). In some cases for quadratic trajectories, when the predicted peak is within the age range, it also indicates a brief developmental period where volume is relatively stable. On the other hand, a linear fit indicates that the rate of change in volume is relatively constant, and does not change direction during 2–8 years. It may also indicate that the region is not imminently approaching peak volume during this age range, as the rate of development is not slowing as would be expected nearing a peak.

We detected earlier volume decreases in sensory regions compared to other brain areas. For example, the postcentral gyrus, the location of the somatosensory cortex (DiGuiseppi & Tadi, 2023), showed volume decreases from 2 years onward, while adjacent regions showed rapid increases in volume. The ability to detect this regional heterogeneity was made possible by the regional specificity of the atlas used in our segmentation pipeline (Huo, Carass, et al., 2016;Klein et al., 2010). The spatiotemporal pattern observed in this study is in concordance with previous studies that characterized cortical gray matter development in the preschool years with similar or better regional specificity (Brown & Jernigan, 2012;Deoni et al., 2015,Remer et al., 2017), although these studies did not use longitudinal data. Our longitudinal analysis confirms that sensory regions show some of the earliest volume decreases, likely in conjunction with early functional/behavioral development of sensory systems.

A limited number of absolute volume regions (8/116) throughout the brain were best fit by a null trajectory. The null fit could have one of several meanings; first, a null fit could indicate that change in volume between 2 and 8 years is incredibly small and/or slow, resulting in the appearance of a null trajectory. Alternatively, if the direction and magnitude of volume change varies considerably between individuals, then a null model may be preferred by the AIC. In our study, AIC weights indicated that null fits were roughly twice as likely to be the best fitting model than the next-best fitting model, which bolsters our confidence in these null trajectories. However, it does not rule out the contribution of intra-individual variability to model selection.

Roughly 20% of regions were best fit by a null model when volume was expressed as a proportion of total ICV. Specifically, this indicates that the region remains the same percent of total ICV throughout the age range. In other words, these regions appear to be increasing in volume at roughly the same rate as ICV. In the case of relatively large frontal areas, such as the right inferior temporal gyrus, left middle frontal gyrus, right and left superior frontal gyrus, these large regions make up a significant portion of total ICV and are thus large contributors to the total ICV trajectory.

An additional type of spatiotemporal variance observed in our study was medium to large age effects in most subcortical structures but not in most frontal cortical regions, particularly superior frontal regions. These larger effect sizes for age in subcortical structures indicate that the variance of the age effect is similar to the total variance of the model. In other words, change in subcortical volumes are well explained by changes in age in the 2–8 year developmental period and less so in superior frontal regions. The disconnect in the developmental pattern between subcortical and superior frontal regions observed here may offer support for the developmental mismatch hypothesis, wherein a mismatch in developmental timing of maturation in subcortical and prefrontal structures is related to developmentally appropriate behaviour (Mills et al., 2021). While this has predominantly been described in adolescents with regard to amygdala-prefrontal cortical development and risk-taking behavior, it stands to reason that a mismatch in fontal and subcortical development in young children may be related to rapidly developing executive functioning, and the networks underlying the development of a central executive (Clark et al., 2021;McKenna et al., 2017;Nachshon et al., 2020).

### 4.2 Absolute increases, proportional decreases

Absolute total gray matter volume showed a non-linear increase during early childhood, with rapid increases early, followed by slower increases, a peak, and slight decreases approaching 8 years. Research in infants has indicated large (100–150%) increases in total gray matter volume over the first year and an additional 14–18% increase over the second year (Fukami-Gartner et al., 2023;Gilmore et al., 2012;Kelly et al., 2023;Knickmeyer et al., 2008;Peterson et al., 2021). Here, we showed moderate increases until approximately 6.5 years (1–4% increase annually), followed by modest decreases (<1% decrease annually).

Rates of volume change were regionally heterogeneous and varied by age; some regions showed large (up to 12%) increases in volume early on while other regions concurrently showed large volume decreases (up to 7%). The regional heterogeneity we observed highlights the importance of using smaller regional units to describe developmental volume changes; some regions decrease while others increase; simply looking at total gray matter volume dilutes developmental effects and is less informative than detailed vertex-, voxel-, or region-wise approaches.

Total proportional gray matter volume decreased between 2 and 8 years of age, as did the majority of individual regions when expressed as a percent of ICV. It has previously been understood that proportional decreases reflect concurrent volume increases in other tissue types, such as white matter; myelination of axons is ongoing during childhood (Williamson & Lyons, 2018), and an increase in myelinated axons could change the relative proportion of gray matter (Houdé & Borst, 2022;Natu et al., 2019;Sherin & Bartzokis, 2011;Walhovd et al., 2017). However, emerging evidence suggests that decreases in gray matter volume are not spatiotemporally synchronous with increases in white matter volume, calling the exact nature of this relationship between gray and white matter into question (Chad & Lebel, in review). Nevertheless, our study indicates that while absolute gray matter volume is broadly increasing in early childhood, the proportion of gray matter to ICV is broadly decreasing, suggesting that at least one other tissue type contributing to ICV is increasing in volume more rapidly than gray matter.

### 4.3 Few sex differences in trajectories

Our finding of larger absolute volume in males compared to females is consistent with widely accepted scientific consensus across previous studies (seeDeCasien et al., 2022;Kaczkurkin et al., 2019;Ruigrok et al., 2014for an overview of relevant studies). The main effects of sex were tempered when the volume was normalized by ICV, which is not surprising given the known relationship between sex and ICV.

Notably, none of the regional volumes expressed in absolute terms (mm^3^) had even nominally significant age-by-sex or age^2^-by-sex interaction effects. For regional volume expressed as percent of ICV, only 2 regional volumes had a significant age-by-sex interactions after FDR correction. An additional 5 regions expressed as percent ICV had nominally significant age-by-sex interactions. As these 7 regions only represent 6% of the regions we examined, our conclusion remains that few sex differences in development trajectories are present between ages 2 and 8 years. Previous research in neonates suggested that growth rates for total brain volume differ by sex, with male brain volume increasing more rapidly than female brain volume (Holland et al., 2014), resulting in male brain volume being larger than that of females. In samples of children spanning the typical pubertal period, it has similarly been found that rates of regional gray matter volume development differ between males and females over the age of 8; Gennatas et al. found that caudate, and frontal and temporal gray matter volume increase more rapidly in males until about age 15 years, after which rates of volume change become more similar for males and females (2017). Additionally, they observed that rates of thalamic and insular volume change were consistently larger in males than females through age 23 years, while parietal and occipital volume change was initially larger in males before females showed increased rates of change after age 15. Only in the putamen did the study find that females had a faster rate of volume change than males (Gennatas et al., 2017). To our knowledge, no previous study has specifically reported sex differences in development rates in the occipital fusiform gyrus or medial postcentral gyrus; however, this is likely due to differences in the atlases used for segmentation or ways in which segmentations are combined for analysis. What is clear, however, is that sex differences in rates of development appear to be global in infancy and later childhood and adolescence (Gennatas et al, 2017;Holland et al., 2014;Lenroot et al., 2007), while in our study we found no sex differences in trajectories for either total gray matter volume and only few differences in regional volume development. This could be explained by the fact that the perinatal period and puberty (but not early childhood) are associated with surges in gonadal hormones, leading to brain sex differences (Breedlove et al., 2010,Sisk et al., 2013). That the medial postcentral gyrus and occipital fusiform gyrus show sex differences in development during the early childhood period suggests that these differences are maintained in these regions, even in the absence of an active surge in gonadal hormones.

Sex differences in human brain structure observed via MRI may stem from a myriad of factors, including differential exposure to sex hormones*in utero*, perinatally, and during puberty, sex chromosome complement, or through interactions with environmental exposures (DeCasien et al., 2022;Giedd et al., 2006,^2012^;Joel et al., 2015;Marrocco & McEwen, 2016;McCarthy, 2016). Given that previous findings in later childhood and adolescence showed sex-differentiated developmental gray matter volume trajectories, we asked whether this also happens during an earlier developmental period and expected to find widespread differences in rates of development between males and females. However, we found no sex differences in absolute volume trajectories, and only two regions (left medial postcentral gyrus and right occipital fusiform gyrus) showed sex differences in proportional volume trajectories with females having steeper slopes than males. Previous studies of later childhood and adolescence have indicated earlier volume peaks for females (Lenroot et al., 2007;Tanaka et al., 2012;Uematsu et al., 2012), suggesting sex-dependent timing of developmental processes such as synaptogenesis and pruning. However, these differences do not appear to be present before age 8 years. Our findings suggest that while rates of gray matter development can vary by sex, developmental sex differences in early childhood are less extensive than in older childhood and adolescence.

Again, a key difference between our study and prior work is the age range of the samples. Particularly, given the young age of our sample (the oldest subject was scanned at age 8.04 years), it is likely that all subjects are prepubertal, though it should be noted that this was not measured directly. The young age range of our sample sets it apart from previous longitudinal studies of gray matter development, which found developmental sex differences in longitudinal samples with age ranges spanning from childhood through adolescence and adulthood (Lenroot et al., 2007;Tanaka et al., 2012;Uematsu et al., 2012). Previous studies have characterized the relationship between brain structure and puberty-related hormones, such as testosterone and estradiol, and found that gray matter volume is related to gonadal hormone levels, even when accounting for age-related volume effects, though few have examined this relationship longitudinally (seePeper et al., 2011for a review). The relative lack of sex differences found in our early childhood sample suggests that the onset of puberty, and associated changes in gonadal hormones, may promote the emergence of wider-spread sex differences in gray matter volume, whereas the few regions with developmental sex differences identified in this study may represent brain sex differences preserved from the perinatal hormonal surge. The longitudinal data and the large number of individual scans included in our study provide confidence that the lack of sex differences found in this study is not due to a lack of power, but rather that limited sex differences in gray matter development in early childhood may be a biological reality. Further longitudinal studies of brain development, which measure and adequately account for the effects of puberty, are needed to further elucidate the mechanisms underlying sexual differentiation in the human brain.

### 4.4 Study limitations

Our sample is a primarily white, high-income, high education sample that is not representative of the Canadian population as a whole (Statistics Canada, 2021). Care should be taken in extrapolating these findings to samples with different demographic makeups, not only because socioeconomic status is related to trajectories of brain development (Tooley et al., 2021), but also because misapplication of findings from a majority white sample to different ethnic groups or assuming that a majority white sample is representative of the broader population may inadvertently perpetuate racism in biomedical and neuroscientific research (Gilpin & Taffe, 2021;Henrich et al., 2010). Next, movement artifacts are common in pediatric neuroimaging studies. However, our lab has developed best practices for scanning un-sedated young children, which support the success rate of scans (Thieba et al., 2018). Nonetheless, head motion artifacts are inevitably present in our dataset, so we visually inspected all T1-weighted images and excluded those scans with excessive movement. Furthermore, most processing and analysis pipelines for segmenting and measuring gray matter volume are developed on adult brain MRI data, which does not always translate to robust segmentations in pediatric MRI. The software we used to segment gray matter volumes, MaCRUISE, was likewise developed on adult brain data. However, the machine-learning approach to multi-atlas segmentation combined with internally consistent segmentations and surfaces yielded robust volume measurement in our sample of young, typically developing children (Bermudez et al., 2020;Huo, Plassard, et al., 2016). To ensure a high quality of segmentations, all processed images were visually inspected by trained raters. In the case of outlier values, segmented images were double-checked to ensure that only biologically plausible segmentations are included in the analysis. A second potential limitation of MaCRUISE is that the pipeline does not account for the repeated nature of scans, a feature available in other comparable pipelines such as Freesurfer. Given that MaCRUISE yielded far more robust segmentations in our pediatric data than Freesurfer, we considered use of MaCRUISE despite the lack of a longitudinal pipeline to be a worthy trade off. Additionally, we visually inspected spaghetti plots of raw volume values to ensure biological plausibility of intra-individual change between scanning sessions which would have ameliorated any significant issues posed by the lack of a longitudinal segmentation pipeline. We also acknowledge some of the limitations associated with quadratic trajectories, some of which could be addressed by more complex model fits such as spline or localized regression approaches. During preparation of this manuscript, we visualized the regional volume data over time using a localized regression (LOESS) model and observed that the shape of the LOESS fit did not differ much from the linear or quadratic fits in our data. Additionally, deriving metrics such as peak age and percent change can be non-trivial for splines, GAMMs, and other non-linear approaches, although this may be changing given the advent of new approaches such as linear estimation with non-linear inference (LENI;McCormick, 2024, OSF Preprint). In this trade-off between flexibility and interpretability, we sided with interpretability and utilized quadratic models as the most advanced fit. Lastly, our study only focused on gray matter volume, which is a composite of two more specific measurements of the cortex, surface area, and cortical thickness. For example, an increase in gray matter volume may be attributable to an increase in cortical thickness, surface area, or both. MaCRUISE provides region-based estimates of cortical thickness (i.e., mean thickness) and surface area; however, for surface-based measurements, a vertex-wise approach is preferred to a region of interest approach; intra-regional variation in cortical thickness (Carey et al., 2018) is lost when averaged across a region of interest, as are changes in cortical thickness that cross regional anatomical boundaries (Backhausen et al., 2022). Future neuroimaging studies of brain development should report vertex-wise measurement of cortical thickness and surface area whenever possible.

## 5 Conclusions

Childhood is a key developmental period, and this study provides a detailed characterization of gray matter volume changes in young children, showing regional patterns consistent with studies in older children, but little evidence for sex differences in development rates at this young age. Our results constitute a basis for comparison of brain development in various clinical samples, where brain development patterns may differ.

## Supplementary Material

Supplementary Material

## Data Availability

MRI data are available through the Public Repository Open Science Framework DOI 10.17605/OSF.IO/AXZ5Rhttps://osf.io/axz5r/. Code is available by request.
