# Supplementary material for: Few sex differences in regional gray matter volume growth trajectories across early childhood
Source: Imaging Neurosci (Camb). 2024 May 20;2:imag-2-00154. doi: 10.1162/imag_a_00154 (PMC12247606; doi:10.1162/imag_a_00154)
Supplement: Supplementary Material [file imag_a_00154-supp.pdf]

Table S1. AIC values for model selection in all regions, expressed as absolute volume (mm^3) and proportional volume (%ICV)

	mm^3			%ICV		
	Null	Linear	Quadratic	Null	Linear	Quadratic
Total Gray Matter	9444.96	9340.48	**9313.40**	1458.19	1440.11	**1435.26**
Right Accumbens Area	4342.83	4324.38	**4311.65**	-3135.26	-3133.75	**-3138.18**
Left Accumbens Area	4351.47	4326.25	**4307.96**	-3154.62	-3152.63	**-3161.54**
Right Amygdala	4610.65	4548.23	**4536.92**	-2975.87	-2978.35	**-2980.89**
Left Amygdala	4605.49	4533.73	**4516.27**	-2976.51	-2979.59	**-2985.06**
Right Caudate	5359.97	5285.52	**5263.60**	-2197.75	**-2230.28**	-2229.27
Left Caudate	5379.69	5256.19	**5224.40**	-2243.07	-2255.49	**-2257.16**
Right Hippocampus	5473.05	5351.05	**5317.99**	-2150.29	-2150.83	**-2158.42**
Left Hippocampus	5414.17	5268.81	**5231.26**	-2144.45	-2145.94	**-2152.06**
Right Pallidum	4704.74	**4596.75**	4596.83	-2810.41	**-2814.55**	-2813.51
Left Pallidum	4686.64	**4606.31**	4608.06	-2813.43	-2811.53	**-2817.67**
Right Putamen	5755.91	5683.08	**5642.78**	-1838.36	-1837.79	**-1851.89**
Left Putamen	5760.36	5654.40	**5591.24**	-1880.61	-1878.61	**-1906.96**
Right Thalamus Proper	5942.33	5742.94	**5714.09**	-1792.82	-1796.84	**-1799.35**
Left Thalamus Proper	5991.78	5751.20	**5707.25**	-1751.19	-1760.32	**-1766.53**
Right Ventral DC	5567.22	5148.56	**5138.00**	-2134.09	**-2242.38**	-2240.81
Left Ventral DC	5616.06	5174.66	**5173.62**	-2102.03	-2228.17	**-2230.68**
Right Basal Forebrain	4484.55	**4485.36**	4485.78	-3002.43	**-3030.27**	-3028.74
Left Basal Forebrain	4623.94	4624.27	**4616.47**	-2871.23	**-2890.28**	-2890.24
Right Anterior Cingulate Gyrus	6216.00	6182.89	**6181.90**	**-1314.80**	-1313.08	-1311.09
Left Anterior Cingulate Gyrus	6069.34	6028.94	**6010.59**	-1494.97	-1501.67	**-1503.07**
Right Anterior Insula	5938.24	5724.04	**5678.17**	-1705.72	-1773.32	**-1792.42**
Left Anterior Insula	5991.62	5816.38	**5779.86**	-1661.10	-1713.48	**-1729.48**
Right Anterior Orbital Gyrus	5656.93	5654.70	**5653.00**	-1821.57	**-1827.89**	-1826.59

Left Anterior Orbital Gyrus	5496.02	**5487.72**	5488.46	-2017.26	**-2019.99**	-2017.99
Right Angular Gyrus	6669.12	6626.96	**6606.98**	-948.05	-949.58	**-954.39**
Left Angular Gyrus	6700.44	6663.89	**6653.99**	-837.77	-836.08	**-838.11**
Right Calcarine Cortex	6297.29	6296.36	**6275.78**	-1148.34	-1148.02	**-1162.17**
Left Calarine Cortex	6195.63	6192.28	**6184.25**	-1231.52	-1232.61	**-1235.09**
Right Central Operculum	5769.50	**5756.25**	5758.24	-1750.27	-1769.50	**-1773.06**
Left Central Operculum	5809.71	**5772.18**	5774.15	-1806.85	-1812.20	**-1817.82**
Right Cuneus	6085.31	6086.11	**6082.23**	-1355.40	**-1423.36**	-1421.55
Left Cuneus	6093.69	6091.33	**6088.91**	-1332.28	**-1414.21**	-1412.21
Right Entorhinal Area	5470.15	5423.11	**5404.91**	-2033.71	-2040.25	**-2049.59**
Left Entorhinal Area	5391.42	5349.28	**5332.85**	-2122.61	-2127.08	**-2136.51**
Right Frontal Operculum	5494.20	5443.22	**5440.84**	**-2063.68**	-2062.41	-2060.78
Left Frontal Operculum	5527.39	**5488.49**	5489.94	**-1975.27**	-1973.31	-1972.38
Right Frontal Pole	6106.18	6057.50	**6049.09**	-1439.50	-1440.83	**-1443.67**
Left Frontal Pole	6119.20	6097.49	**6081.33**	-1421.15	-1419.18	**-1428.05**
Right Fusiform Gyrus	6362.26	6259.62	**6239.06**	-1284.48	-1294.21	**-1302.16**
Left Fusiform Gyrus	6299.67	6189.69	**6152.57**	-1340.27	-1345.20	**-1360.50**
Right Gyrus Rectus	5829.88	**5806.17**	5807.69	-1666.21	**-1666.93**	-1665.07
Left Gyrus Rectus	5736.69	**5707.75**	5709.00	-1760.02	**-1762.28**	-1760.34
Right Inferior Occipital Gyrus	6517.47	6513.76	**6503.82**	-989.11	-995.67	**-998.13**
Left Inferior Occipital Gyrus	6537.44	6536.03	**6530.64**	-944.98	**-957.43**	-956.26
Right Inferior Temporal Gyrus	6704.34	6658.52	**6650.64**	**-851.87**	-850.07	-851.77
Left Inferior Temporal Gyrus	6624.37	6542.51	**6531.18**	-1003.19	-1006.27	**-1008.59**
Right Lingual Gyrus	6600.06	6589.58	**6579.31**	-935.05	-939.94	**-941.07**
Left Lingual Gyrus	6470.86	6444.95	**6440.06**	-1095.45	**-1097.51**	-1096.12
Right Lateral Orbital Gyrus	5794.96	5779.95	**5776.67**	**-1707.64**	-1705.65	-1705.92
Left Lateral Orbital Gyrus	5823.85	5823.75	**5821.28**	-1677.43	**-1686.85**	-1685.46
Right Middle Cingulate Gyrus	5968.92	**5960.49**	5959.76	-1545.85	**-1563.16**	-1561.25
Left Middle Cingulate Gyrus	**6186.23**	6187.77	6189.04	-1284.55	**-1324.91**	-1323.93

Right Medial Frontal Cortex	5573.47	5539.03	**5530.61**	**-1960.22**	-1958.42	-1958.81
Left Medial Frontal Cortex	5463.89	5439.40	**5433.86**	**-2033.60**	-2032.82	-2032.20
Right Middle Frontal Gyrus	7141.47	7042.09	**7020.23**	-601.84	-601.46	**-607.59**
Left Middle Frontal Gyrus	7210.71	7152.91	**7144.90**	**-495.02**	-493.02	-492.35
Right Middle Occipital Gyrus	6207.59	6209.55	**6200.80**	-1228.05	-1271.61	**-1271.66**
Left Middle Occipital Gyrus	6252.56	6253.50	**6249.56**	-1211.57	**-1243.96**	-1242.53
Right Medial Orbital Gyrus	6067.85	6049.28	**6048.79**	**-1454.73**	-1452.81	-1451.08
Left Medial Orbital Gyrus	6069.46	6054.50	**6050.43**	**-1445.80**	-1443.81	-1443.51
Right Postcentral Gyrus Medial Segment	5515.40	5500.52	**5496.58**	-1889.55	-1935.72	**-1945.76**
Left Postcentral Gyrus Medial Segment	5480.86	5474.47	**5472.27**	-1915.60	-1950.34	**-1958.88**
Right Precentral Gyrus Medial Segment	**5711.37**	5712.51	5712.24	-1739.89	-1779.07	**-1787.27**
Left Precentral Gyrus Medial Segment	5635.11	5636.74	**5633.64**	-1817.21	-1863.41	**-1880.06**
Right Superior Frontal Gyrus Medial Segment	6400.57	6372.93	**6370.46**	-1188.61	**-1192.01**	-1190.11
Left Superior Frontal Gyrus Medial Segment	6286.36	6226.26	**6215.55**	**-1328.95**	-1327.12	-1326.58
Right Middle Temporal Gyrus	6861.46	6738.69	**6696.31**	-795.72	-801.83	**-819.52**
Left Middle Temporal Gyrus	6804.15	6709.23	**6679.04**	-860.95	-860.13	**-869.09**
Right Occipital Pole	6216.84	**6215.52**	6216.24	**-1219.92**	-1218.02	-1216.23
Left Occipital Pole	6364.23	**6341.14**	6343.13	-1089.39	**-1095.64**	-1094.06
Right Occipital Fusiform Gyrus	**6153.38**	6154.46	6156.41	-1331.53	**-1373.10**	-1372.07
Left Occipital Fusiform Gyrus	6136.44	**6135.93**	6137.28	-1316.21	**-1365.18**	-1364.18
Right Opercular Part of the Inferior Frontal Gyrus	5919.71	5886.70	**5879.18**	**-1595.62**	-1594.55	-1594.77
Left Opercular Part of the Inferior Frontal Gyrus	5965.83	5947.38	**5942.44**	**-1521.47**	-1521.38	-1520.64
Right Orbital Part of the Inferior Frontal Gyrus	5640.77	5606.44	**5601.66**	-1854.42	-1854.69	**-1855.02**
Left Orbital Part of the Inferior Frontal Gyrus	5651.96	5629.35	**5628.90**	**-1855.74**	-1854.05	-1852.40
Right Posterior Cingulate Gyrus	5972.11	5918.34	**5910.05**	**-1645.84**	-1644.20	-1644.23
Left Posterior Cingulate Gyrus	5929.70	5905.05	**5899.45**	-1663.80	**-1683.15**	-1681.33
Right Precuneus	6617.29	6579.65	**6569.76**	-997.30	-1003.36	**-1003.42**
Left Precuneus	6553.67	6542.08	**6533.39**	-1012.06	**-1054.98**	-1053.63

Right Parahippocampal Gyrus	5760.76	5737.59	**5734.05**	**-1727.13**	-1725.15	-1723.94
Left Parahippocampal Gyrus	5861.98	5838.66	**5829.46**	-1626.53	-1624.53	**-1626.59**
Right Posterior Insula	5746.60	5647.74	**5635.39**	-1778.55	-1809.62	**-1815.04**
Left Posterior Insula	5445.07	5348.42	**5318.08**	-2146.25	-2149.38	**-2157.97**
Right Parietal Operculum	5588.12	5564.95	**5563.70**	**-1907.09**	-1907.06	-1905.10
Left Parietal Operculum	5630.50	**5574.00**	5575.96	-1879.58	-1877.99	**-1880.76**
Right Postcentral Gyrus	6812.04	6811.94	**6810.97**	-668.03	-711.48	**-720.19**
Left Postcentral Gyrus	**6909.77**	6911.66	6913.65	-548.87	-579.38	**-579.58**
Right Posterior Orbital Gyrus	5675.50	5651.90	**5640.17**	-1849.59	-1850.76	**-1852.15**
Left Posterior Orbital Gyrus	5759.15	5725.51	**5716.41**	-1793.06	-1791.73	**-1793.07**
Right Planum Polare	5431.49	5401.19	**5388.48**	-2079.70	-2077.70	**-2082.66**
Left Planum Polare	5378.70	5342.78	**5334.67**	**-2175.78**	-2175.09	-2174.77
Right Precentral Gyrus	6669.10	**6665.05**	6666.59	-898.76	**-931.16**	-930.81
Left Precentral Gyrus	6703.83	**6689.68**	6690.70	-870.29	**-881.50**	-880.51
Right Planum Temporale	5503.50	5493.28	**5492.86**	-1984.78	**-1988.45**	-1986.49
Left Planum Temporale	5569.94	5556.23	**5554.63**	-1964.14	**-1968.43**	-1966.45
Right Subcallosal Area	5236.96	5230.59	**5226.18**	-2278.25	-2279.33	**-2279.64**
Left Subcallosal Area	5280.94	5275.76	**5273.14**	**-2229.67**	-2229.42	-2229.28
Right Superior Frontal Gyrus	7012.61	**6989.27**	6991.22	**-597.93**	-597.27	-597.29
Left Superior Frontal Gyrus	7058.03	**7035.93**	7037.75	**-513.29**	-511.46	-510.44
Right Supplementary Motor Cortex	6134.15	**6127.30**	6129.11	-1403.60	-1411.42	**-1413.81**
Left Supplementary Motor Cortex	6201.19	**6173.18**	6175.12	**-1333.51**	-1331.51	-1330.65
Right Supramarginal Gyrus	6617.03	6589.62	**6567.43**	-900.73	-900.33	**-909.51**
Left Supramarginal Gyrus	6719.80	6705.31	**6700.34**	**-795.00**	-794.69	-794.22
Right Superior Occipital Gyrus	6208.14	6202.68	**6195.74**	-1246.48	-1249.58	**-1250.79**
Left Superior Occipital Gyrus	6180.94	**6180.91**	6181.78	-1270.61	**-1280.31**	-1278.55
Right Superior Parietal Lobule	**7001.66**	7002.21	7003.71	-476.46	-506.23	**-507.05**
Left Superior Parietal Lobule	**6930.07**	6932.06	6933.50	-566.16	**-586.79**	-584.91
Right Superior Temporal Gyrus	6391.53	6367.05	**6357.20**	-1161.94	-1168.43	**-1169.12**

Left Superior Temporal Gyrus	6467.38	6426.23	**6409.96**	-1106.07	-1104.09	**-1111.26**
Right Temporal Pole	6444.91	6301.01	**6270.66**	-1148.52	-1187.02	**-1203.55**
Left Temporal Pole	6501.76	6389.69	**6371.64**	-1090.48	-1122.40	**-1131.05**
Right Triangular Part of the Inferior Frontal Gyrus	6138.10	6110.69	**6074.91**	-1408.51	-1406.78	**-1427.81**
Left Triangular Part of the Inferior Frontal Gyrus	6138.46	6089.10	**6088.47**	-1397.14	**-1397.17**	-1395.19
Right Transverse Temporal Gyrus	**5380.71**	5382.18	5383.77	-2044.04	-2076.32	**-2077.50**
Left Transverse Temporal Gyrus	5333.13	**5319.56**	5321.53	-2158.10	-2165.66	**-2168.47**

Table S2. Final model summaries for absolute volume trajectories. All regional models were reduced to only include a main effect of sex.

Region		mm^3				
		Beta	SE	η_p^2	p	q
Total Gray Matter	Intercept	546653.776	17825.159		<0.001	<0.001
	Age	47660.502	6387.09	0.163		
	Sex	70360.653	11364.582	0.236		
	Age^2	-3485.186	616.571	0.101		
Right Accumbens Area	Intercept		34.718		0.092	0.102
	Age	62.077	13.466	0.063		
	Sex	22.644	13.335	0.023		
	Age^2	-5.119	1.306	0.048		
Left Accumbens Area	Intercept	316.707	34.568		0.012	0.0152
	Age	73.216	13.44	0.086		
	Sex	32.977	12.935	0.05		
	Age^2	-6.046	1.304	0.065		
Right Amygdala	Intercept	538.369	45.124		<0.001	<0.001
	Age	88.879	17.421	0.077		
	Sex	72.925	18.187	0.115		
	Age^2	-6.389	1.688	0.045		
Left Amygdala	Intercept	517.359	44.383		<0.001	<0.001
	Age	103.824	17.258	0.103		
	Sex	74.136	16.594	0.141		
	Age^2	-7.669	1.674	0.064		
Right Caudate	Intercept	3294.577	106.182		0.319	0.325
	Age	233.494	36.129	0.13		
	Sex	79.648	79.633	0.008		
	Age^2	-17.448	3.485	0.083		
Left Caudate	Intercept	3177.188	101.185		0.523	0.528
	Age	263.406	33.009	0.187		
	Sex	53.458	83.379	0.003		
	Age^2	-19.068	3.183	0.115		
Right Hippocampus	Intercept	2512.364	116.463		<0.001	0.001
	Age	354.047	43.316	0.187		
	Sex	215.851	62.27	0.09		
	Age^2	-25.855	4.186	0.117		
Left Hippocampus	Intercept	2418.072	103.958		0.009	0.012
	Age	336.968	38.251	0.211		
	Sex	157.309	58.923	0.054		
	Age^2	-24.279	3.695	0.131		

Right Putamen	Intercept	3466.9	173.499		<0.001	0.001
	Age	524.109	63.321	0.193		
	Sex	354.015	102.312	0.089		
	Age^2	-41.61	6.115	0.14		
Left Putamen	Intercept	3322.805	160.91		0.01	0.014
	Age	594.957	56.693	0.28		
	Sex	284.066	109.003	0.052		
	Age^2	-47.034	5.471	0.208		
Right Thalamus Proper	Intercept	5509.684	188.564		<0.001	<0.001
	Age	579.842	68.179	0.201		
	Sex	428.635	115.908	0.098		
	Age^2	-37.951	6.583	0.104		
Left Thalamus Proper	Intercept	5572.759	185.305		0.011	0.014
	Age	640.107	62.003	0.275		
	Sex	375.645	144.726	0.051		
	Age^2	-42.434	5.98	0.153		
Right Ventral Diencephalon	Intercept	2931.853	89.619		<0.001	<0.001
	Age	275.418	32.09	0.205		
	Sex	251.199	57.294	0.134		
	Age^2	-11.261	3.098	0.045		
Left Ventral Diencephalon	Intercept	3144.908	94.099		<0.001	<0.001
	Age	231.905	33.565	0.143		
	Sex	234.329	61.037	0.106		
	Age^2	-5.811	3.24	0.011		
Left Basal Forebrain	Intercept	565.516	50.165		<0.001	<0.001
	Age	69.214	19.656	0.037		
	Sex	109.395	17.111	0.248		
	Age^2	-6.344	1.909	0.034		
Right Anterior Cingulate Gyrus	Intercept	4491.723	350.691		<0.001	<0.001
	Age	363.877	132.389	0.025		
	Sex	827.687	170.51	0.16		
	Age^2	-22.94	12.804	0.011		
Left Anterior Cingulate Gyrus	Intercept	4579.456	265.347		<0.001	<0.001
	Age	525.894	91.975	0.104		
	Sex	1100.319	189.165	0.215		
	Age^2	-41.39	8.874	0.072		
Right Anterior Insula	Intercept	2175.32	175.356		<0.001	<0.001
	Age	634.33	61.813	0.271		
	Sex	638.723	118.592	0.19		
	Age^2	-43.605	5.965	0.16		

Left Anterior Insula	Intercept	2271.439	201.404		<0.001	<0.001
	Age	663.163	73.131	0.222		
	Sex	692.021	121.548	0.206		
	Age^2	-46.011	7.062	0.13		
Right Anterior Orbital Gyrus	Intercept	2050.531	187.349		0.003	0.004
	Age	167.611	72.156	0.017		
	Sex	233.426	77.298	0.066		
	Age^2	-14.03	6.99	0.013		
Right Angular Gyrus	Intercept	10615.689	601.142		<0.001	<0.001
	Age	1339.696	225.526	0.107		
	Sex	1311.731	304.835	0.129		
	Age^2	-106.12	21.804	0.075		
Left Angular Gyrus	Intercept	9115.149	661.387		<0.001	<0.001
	Age	1161.901	253.762	0.064		
	Sex	1053.126	282.671	0.101		
	Age^2	-88.154	24.574	0.041		
Right Calcarine Cortex	Intercept	1510.049	415.238		0.007	0.009
	Age	821.752	160.084	0.079		
	Sex	470.067	169.711	0.06		
	Age^2	-76.053	15.51	0.074		
Left Calcarine Cortex	Intercept	2979.529	362.824		0.115	0.124
	Age	489.654	137.689	0.041		
	Sex	269.554	169.778	0.02		
	Age^2	-42.925	13.321	0.034		
Right Cuneus	Intercept	5163.051	306.136		<0.001	<0.001
	Age	269.105	113.789	0.019		
	Sex	671.598	164.272	0.121		
	Age^2	-27.992	10.996	0.022		
Left Cuneus	Intercept	5182.271	309.062		<0.001	<0.001
	Age	215.878	115.487	0.012		
	Sex	725.778	160.713	0.142		
	Age^2	-24.583	11.163	0.016		
Right Entorhinal Area	Intercept	730.082	144.471		0.002	0.003
	Age	330.285	57.134	0.09		
	Sex	135.827	43.39	0.075		
	Age^2	-25.873	5.561	0.062		
Left Entorhinal Area	Intercept	859.322	130.896		0.008	0.011
	Age	282.742	51.548	0.083		
	Sex	112.684	41.748	0.056		
	Age^2	-22.199	5.012	0.057		

Right Frontal Operculum	Intercept	1809.991	135.285		0.001	0.002
	Age	166.545	49.893	0.037		
	Sex	255.367	75.771	0.085		
	Age^2	-10.408	4.82	0.016		
Right Frontal Pole	Intercept	3089.26	301.217		0.063	0.072
	Age	500.258	113.123	0.062		
	Sex	284.277	151.719	0.027		
	Age^2	-35.948	10.937	0.036		
Left Frontal Pole	Intercept	2312.546	315.22		<0.001	<0.001
	Age	621.717	120.065	0.081		
	Sex	553.448	143.332	0.106		
	Age^2	-51.316	11.619	0.061		
Right Fusiform Gyrus	Intercept	4890.392	371.979		<0.001	<0.001
	Age	935.13	138.979	0.133		
	Sex	977.912	193.585	0.169		
	Age^2	-66.49	13.434	0.078		
Left Fusiform Gyrus	Intercept	5082.068	329.653		<0.001	<0.001
	Age	1031.93	122.052	0.198		
	Sex	954.901	180.813	0.187		
	Age^2	-77.453	11.793	0.131		
Right Inferior Occipital Gyrus	Intercept	5674.357	542.244		<0.001	<0.001
	Age	837.925	208.52	0.05		
	Sex	1116.169	227.013	0.164		
	Age^2	-73.799	20.197	0.042		
Left Inferior Occipital Gyrus	Intercept	6123.593	546.355		<0.001	<0.001
	Age	658.939	209.391	0.031		
	Sex	1578.693	235.859	0.258		
	Age^2	-57.932	20.275	0.026		
Right Inferior Temporal Gyrus	Intercept	8441.736	657.088		<0.001	<0.001
	Age	1136.476	253.768	0.06		
	Sex	1361.41	263.951	0.173		
	Age^2	-82.461	24.592	0.035		
Left Inferior Temporal Gyrus	Intercept	8135.64	552.735		<0.001	<0.001
	Age	1129.642	210.502	0.087		
	Sex	1245.524	251.622	0.162		
	Age^2	-77.892	20.371	0.047		
Right Lingual Gyrus	Intercept	7677.922	595.993		<0.001	<0.001
	Age	969.599	229.118	0.055		
	Sex	1258.236	250.232	0.167		
	Age^2	-81.562	22.191	0.043		

Left Lingual Gyrus	Intercept	7645.879	502.772			
	Age	687.152	192.916	0.04		
	Sex	854.44	214.766	0.116	<0.001	<0.001
	Age^2	-50.856	18.682	0.024		
Right Lateral Orbital Gyrus	Intercept	1869.711	221.886			
	Age	253.661	85.588	0.027		
	Sex	116.247	90.211	0.013	0.199	0.208
	Age^2	-19.344	8.293	0.017		
Left Lateral Orbital Gyrus	Intercept	2177.662	228.374			
	Age	209.367	87.2	0.019		
	Sex	333.453	101.773	0.081	0.001	0.002
	Age^2	-18.433	8.44	0.016		
Right Middle Cingulate Gyrus	Intercept	4504.638	266.944			
	Age	230.576	101.717	0.017		
	Sex	619.088	120.992	0.178	<0.001	<0.001
	Age^2	-17.224	9.844	0.01		
Right Medial Frontal Cortex	Intercept	1578.906	155.325			
	Age	253.044	59.044	0.057		
	Sex	311.761	71.751	0.131	<0.001	<0.001
	Age^2	-18.956	5.713	0.036		
Left Medial Frontal Cortex	Intercept	1739.281	135.829			
	Age	181.061	50.333	0.042		
	Sex	146.404	74.154	0.03	0.051	0.058
	Age^2	-13.601	4.863	0.026		
Right Middle Frontal Gyrus	Intercept	18925.309	985.69			
	Age	2494.903	364.277	0.139		
	Sex	3216.517	546.042	0.219	<0.001	<0.001
	Age^2	-179.609	35.195	0.083		
Left Middle Frontal Gyrus	Intercept	19603.782	1185.608			
	Age	2069.025	450.252	0.066		
	Sex	3401.189	551.782	0.234	<0.001	<0.001
	Age^2	-144.266	43.563	0.036		
Right Middle Occipital Gyrus	Intercept	5918.298	359.051			
	Age	458.176	134.218	0.038		
	Sex	694.704	186.267	0.103	<0.001	<0.001
	Age^2	-44.197	12.974	0.039		
Left Middle Occipital Gyrus	Intercept	5891.986	384.04			
	Age	394.872	145.502	0.024		
	Sex	877.221	181.93	0.155	<0.001	<0.001
	Age^2	-36.005	14.075	0.022		

Right Medial Orbital Gyrus	Intercept	2874.44	320.222		0.03	0.035
	Age	293.847	125.048	0.017		
	Sex	250.114	113.889	0.037		
	Age^2	-19.897	12.138	0.008		
Left Medial Orbital Gyrus	Intercept	2714.828	323.708		0.108	0.117
	Age	398.291	126.753	0.029		
	Sex	180.418	111.345	0.02		
	Age^2	-30.91	12.309	0.019		
Right Postcentral Gyrus Medial Segment	Intercept	1773.55	157.526		0.127	0.136
	Age	-186.463	61.226	0.029		
	Sex	90.889	59.158	0.019		
	Age^2	14.395	5.938	0.019		
Left Postcentral Gyrus Medial Segment	Intercept	1630.527	153.296		0.858	0.858
	Age	-149.205	59.605	0.02		
	Sex	-10.253	57.328	0		
	Age^2	11.952	5.781	0.014		
Left Precentral Gyrus Medial Segment	Intercept	3398.559	183.873		0.003	0.004
	Age	-160.06	71.053	0.016		
	Sex	223.028	73.427	0.069		
	Age^2	15.143	6.886	0.016		
Right Superior Frontal Gyrus Medial Segment	Intercept	7490.258	440.761		<0.001	<0.001
	Age	501.36	163.907	0.031		
	Sex	1030.641	235.867	0.133		
	Age^2	-34.694	15.84	0.016		
Left Superior Frontal Gyrus Medial Segment	Intercept	6193.789	360.091		<0.001	<0.001
	Age	659.546	132.971	0.078		
	Sex	853.678	200.328	0.128		
	Age^2	-47.069	12.847	0.045		
Right Middle Temporal Gyrus	Intercept	11285.963	663.929		<0.001	<0.001
	Age	2245.459	247.871	0.218		
	Sex	1811.238	347.11	0.179		
	Age^2	-168.338	23.959	0.145		
Left Middle Temporal Gyrus	Intercept	12095.146	656.571		<0.001	<0.001
	Age	1874.524	245.624	0.165		
	Sex	1476.341	338.989	0.131		
	Age^2	-140.499	23.744	0.107		
Right Opercular Part of the Inferior Frontal Gyrus	Intercept	3510.735	238.928		0.016	0.02
	Age	361.853	88.619	0.055		
	Sex	318.558	129.768	0.048		
	Age^2	-26.981	8.563	0.034		

Left Opercular Part of the Inferior Frontal Gyrus	Intercept	3107.062	256.545			
	Age	319.654	94.321	0.038		
	Sex	409.659	145.993	0.06	0.006	0.008
	Age^2	-24.472	9.111	0.025		
Right Orbital Part of the Inferior Frontal Gyrus	Intercept	1359.391	169.75			
	Age	235.529	64.207	0.043		
	Sex	308.251	81.395	0.103	<0.001	<0.001
	Age^2	-16.813	6.211	0.024		
Left Orbital Part of the Inferior Frontal Gyrus	Intercept	1680.439	178.119			
	Age	161.167	67.534	0.019		
	Sex	196.878	83.913	0.042	0.021	0.025
	Age^2	-10.487	6.533	0.009		
Right Posterior Cingulate Gyrus	Intercept	3449.953	248.097			
	Age	432.742	94.266	0.065		
	Sex	646.808	115.013	0.202	<0.001	<0.001
	Age^2	-30.64	9.121	0.037		
Left Posterior Cingulate Gyrus	Intercept	4640.11	234.161			
	Age	319.421	85.679	0.046		
	Sex	849.823	136.434	0.24	<0.001	<0.001
	Age^2	-24.024	8.275	0.029		
Right Precuneus	Intercept	10712.024	570.145			
	Age	988.858	213.722	0.068		
	Sex	1393.304	290.644	0.159	<0.001	<0.001
	Age^2	-74.734	20.662	0.043		
Left Precuneus	Intercept	11693.403	534.83			
	Age	780.548	196.091	0.052		
	Sex	1421.163	308.569	0.148	<0.001	<0.001
	Age^2	-64.602	18.941	0.039		
Right Parahippocampal Gyrus	Intercept	2090.271	212.29			
	Age	270.217	83.19	0.032		
	Sex	329.116	72.302	0.15	<0.001	<0.001
	Age^2	-19.761	8.08	0.019		
Left Parahippocampal Gyrus	Intercept	2226.095	241.086			
	Age	397.028	94.033	0.053		
	Sex	224.571	86.974	0.052	0.011	0.014
	Age^2	-31.344	9.125	0.037		
Right Posterior Insula	Intercept	1005.742	171.007			
	Age	358.838	63.413	0.099		
	Sex	488.511	92.99	0.183	<0.001	<0.001
	Age^2	-24.098	6.128	0.051		

Left Posterior Insula	Intercept	1620.523	113.417		<0.001	<0.001
	Age	317.561	41.407	0.169		
	Sex	319.674	66.78	0.155		
	Age^2	-23.672	3.999	0.109		
Right Parietal Operculum	Intercept	2051.432	158.15		<0.001	<0.001
	Age	156.651	58.698	0.024		
	Sex	343.913	85.568	0.117		
	Age^2	-10.624	5.672	0.012		
Right Postcentral Gyrus	Intercept	14129.53	841.126		0.008	0.011
	Age	-608.627	328.312	0.011		
	Sex	811.13	300.825	0.058		
	Age^2	53.021	31.864	0.009		
Right Posterior Orbital Gyrus	Intercept	2339.148	173.621		<0.001	<0.001
	Age	297.21	64.402	0.068		
	Sex	414.488	94.252	0.136		
	Age^2	-23.77	6.223	0.048		
Left Posterior Orbital Gyrus	Intercept	2528.914	190.996		<0.001	<0.001
	Age	306.654	70.167	0.062		
	Sex	404.855	109.112	0.1		
	Age^2	-23.192	6.778	0.039		
Right Planum Polare	Intercept	1489.174	133.658		<0.001	<0.001
	Age	252.777	51.612	0.072		
	Sex	203.109	53.758	0.104		
	Age^2	-20	5.001	0.05		
Left Planum Polare	Intercept	1832.431	119.763		0.001	0.002
	Age	190.593	44.812	0.058		
	Sex	208.555	61.764	0.084		
	Age^2	-14.19	4.332	0.036		
Right Planum Temporale	Intercept	1777.003	148.452		0.001	0.001
	Age	123.194	56.214	0.016		
	Sex	241.905	70.606	0.086		
	Age^2	-8.945	5.438	0.009		
Left Planum Temporale	Intercept	1953.875	155.903		<0.001	<0.001
	Age	152.369	58.156	0.023		
	Sex	392.486	81.921	0.158		
	Age^2	-11.222	5.621	0.014		
Right Subcallosal Area	Intercept	918.452	104.788		<0.001	<0.001
	Age	123.174	39.682	0.031		
	Sex	221.493	49.818	0.137		
	Age^2	-10.184	3.839	0.023		

Left Subcallosal Area	Intercept	830.567	112.804			
	Age	115.863	43.106	0.023		
	Sex	216.168	49.942	0.132	<0.001	<0.001
	Age^2	-9.459	4.173	0.017		
Right Supramarginal Gyrus	Intercept	7270.816	585.579			
	Age	1311.247	221.404	0.106		
	Sex	675.062	281.595	0.045	0.018	0.022
	Age^2	-108.631	21.415	0.081		
Left Supramarginal Gyrus	Intercept	7789.166	713.882			
	Age	933.359	277.008	0.035		
	Sex	1119.203	273.01	0.124	<0.001	<0.001
	Age^2	-73.404	26.86	0.024		
Right Superior Occipital Gyrus	Intercept	3772.283	381.999			
	Age	526.317	149.053	0.038		
	Sex	477.311	137.177	0.091	<0.001	0.001
	Age^2	-45.061	14.465	0.03		
Right Superior Temporal Gyrus	Intercept	6986.23	433.937			
	Age	720.625	162.801	0.063		
	Sex	1124.347	220.017	0.179	<0.001	<0.001
	Age^2	-56.812	15.74	0.043		
Left Superior Temporal Gyrus	Intercept	6007.971	477.896			
	Age	1009.519	182.591	0.092		
	Sex	971.257	211.846	0.146	<0.001	<0.001
	Age^2	-79.006	17.675	0.063		
Right Temporal Pole	Intercept	4177.623	398.449			
	Age	1236.414	151.692	0.18		
	Sex	818.413	181.881	0.14	<0.001	<0.001
	Age^2	-87.379	14.679	0.106		
Left Temporal Pole	Intercept	4045.897	455.133			
	Age	1145.995	174.751	0.123		
	Sex	1027.847	193.268	0.184	<0.001	<0.001
	Age^2	-79.405	16.924	0.068		
Right Triangular Part of the Inferior Frontal Gyrus	Intercept	2364.58	319.139			
	Age	896.498	122.856	0.15		
	Sex	456.748	132.272	0.093	<0.001	0.001
	Age^2	-76.623	11.901	0.123		
Left Triangular Part of the Inferior Frontal Gyrus	Intercept	4084.701	323.984			
	Age	348.045	123.742	0.025		
	Sex	249.876	144.04	0.024	0.085	0.096
	Age^2	-19.93	11.978	0.009		

Right Pallidum	Intercept	961.797	20.601			
	Age	34.112	2.906	0.309		
	Sex	36.155	21.347	0.022	0.093	0.102
Left Pallidum	Intercept	1059.415	20.749			
	Age	29	2.939	0.239		
	Sex	49.012	21.423	0.04	0.024	0.029
Left Anterior Orbital Gyrus	Intercept	2036.154	62.966			
	Age	30.248	9.142	0.034		
	Sex	135.296	63.538	0.036	0.035	0.041
Right Central Operculum	Intercept	4204.368	93.66			
	Age	48.602	11.839	0.054		
	Sex	457.786	105.111	0.133	<0.001	<0.001
Left Central Operculum	Intercept	4142.126	99.261			
	Age	77.257	11.632	0.132		
	Sex	562.483	116.005	0.16	<0.001	<0.001
Left Frontal Operculum	Intercept	2376.85	66.976			
	Age	57.622	8.689	0.13		
	Sex	113.296	73.952	0.019	0.128	0.136
Right Gyrus Rectus	Intercept	1840.784	89.294			
	Age	77.235	14.68	0.076		
	Sex	161.851	76.717	0.035	0.037	0.043
Left Gyrus Rectus	Intercept	1845.464	78.685			
	Age	75.102	13.11	0.088		
	Sex	67.258	66.011	0.008	0.31	0.319
Right Occipital Pole	Intercept	2059.606	153.81			
	Age	45.229	24.146	0.011		
	Sex	190.1	141.813	0.015	0.183	0.191
Left Occipital Pole	Intercept	1620.954	179.658			
	Age	146.807	28.192	0.078		
	Sex	391.313	165.734	0.044	0.02	0.024
Left Occipital Fusiform Gyrus	Intercept	5243.599	140.385			
	Age	-29.426	20.742	0.006		
	Sex	571.029	139.171	0.119	<0.001	<0.001
Left Parietal Operculum	Intercept	2512.089	78.773			
	Age	73.072	8.927	0.19		
	Sex	439.264	93.468	0.154	<0.001	<0.001
Right Precentral Gyrus	Intercept	13131.583	269.675			
	Age	108.704	39.56	0.024		
	Sex	1695.764	269.33	0.244	<0.001	<0.001
Left Precentral Gyrus	Intercept	12937.427	282.049			
	Age	178.985	41.929	0.055		

	Sex	1253.989	277.797	0.142	<0.001	<0.001
Right Superior Frontal Gyrus	Intercept	15057.611	405.618			
	Age	334.877	62.868	0.081		
	Sex	1663.332	380.359	0.133	<0.001	<0.001
Left Superior Frontal Gyrus	Intercept	15510.996	431.367			
	Age	350.986	68.241	0.076		
	Sex	1203.673	393.488	0.073	0.003	0.004
Right Supplementary Motor Cortex	Intercept	5115.107	137.155			
	Age	66.018	20.911	0.03		
	Sex	506.251	131.271	0.106	<0.001	<0.001
Left Supplementary Motor Cortex	Intercept	4880.33	148.755			
	Age	125.734	22.039	0.095		
	Sex	380.16	147.04	0.053	0.011	0.014
Left Superior Occipital Gyrus	Intercept	4636.147	149.846			
	Age	34.267	22.196	0.008		
	Sex	441.413	148.15	0.067	0.004	0.005
Left Transverse Temporal Gyrus	Intercept	1739.55	62.263			
	Age	25.041	6.161	0.055		
	Sex	306.664	77.577	0.113	<0.001	<0.001

Table S3. Final model summaries for proportional volume trajectories. Most regional models were reduced to only include a main effect of sex

Region		%ICV				
		Beta	SE	η_p^2	p	q
Total Gray	Intercept	56.4706	0.9234			
	Age	0.6792	0.3630	0.0109		
	Sex	-0.0645	0.3019	0.0004	0.831	0.891
	Age^2	-0.0926	0.0353	0.0219		
Right Accumbens Area	Intercept	0.0375	0.0027			
	Age	0.0025	0.0011	0.0169		
	Sex	-0.0023	0.0009	0.0511	0.013	0.067
	Age^2	-0.0003	0.0001	0.0195		
Left Accumbens Area	Intercept	0.0351	0.0027			
	Age	0.0034	0.0011	0.0312		
	Sex	-0.0016	0.0008	0.0330	0.051	0.179
	Age^2	-0.0003	0.0001	0.0326		
Right Amygdala	Intercept	0.0585	0.0034			
	Age	0.0032	0.0013	0.0176		
	Sex	-0.0007	0.0011	0.0033	0.539	0.740
	Age^2	-0.0003	0.0001	0.0139		
Left Amygdala	Intercept	0.0580	0.0034			
	Age	0.0041	0.0013	0.0279		
	Sex	-0.0007	0.0010	0.0039	0.520	0.725
	Age^2	-0.0004	0.0001	0.0231		
Left Caudate	Intercept	0.3279	0.0072			
	Age	0.0031	0.0025	0.0055		
	Sex	-0.0276	0.0053	0.1803	<.001	<.001
	Age^2	-0.0004	0.0002	0.0126		
Right Hippocampus	Intercept	0.2701	0.0088			
	Age	0.0109	0.0033	0.0351		
	Sex	-0.0111	0.0043	0.0523	0.010	0.057
	Age^2	-0.0010	0.0003	0.0314		
Left Hippocampus	Intercept	0.2600	0.0089			
	Age	0.0103	0.0034	0.0298		
	Sex	-0.0147	0.0041	0.0955	<.001	0.007
	Age^2	-0.0009	0.0003	0.0257		

Left Pallidum	Intercept	0.1108	0.0040			
	Age	-0.0045	0.0015	0.0264		
	Sex	-0.0057	0.0015	0.1068	<.001	0.003
	Age^2	0.0004	0.0001	0.0280		
Right Putamen	Intercept	0.3721	0.0129			
	Age	0.0183	0.0048	0.0473		
	Sex	-0.0115	0.0068	0.0233	0.092	0.281
	Age^2	-0.0019	0.0005	0.0539		
Left Putamen	Intercept	0.3621	0.0117			
	Age	0.0236	0.0043	0.0976		
	Sex	-0.0174	0.0071	0.0464	0.016	0.078
	Age^2	-0.0023	0.0004	0.1010		
Right Thalamus Proper	Intercept	0.5802	0.0136			
	Age	0.0123	0.0050	0.0200		
	Sex	-0.0242	0.0074	0.0793	0.001	0.015
	Age^2	-0.0010	0.0005	0.0149		
Left Thalamus Proper	Intercept	0.5886	0.0138			
	Age	0.0164	0.0049	0.0375		
	Sex	-0.0299	0.0091	0.0796	0.001	0.015
	Age^2	-0.0013	0.0005	0.0278		
Left Ventral Diencephalon	Intercept	0.3304	0.0080			
	Age	-0.0006	0.0030	0.0001		
	Sex	-0.0147	0.0038	0.1122	<.001	0.067
	Age^2	0.0007	0.0003	0.0166		
Left Anterior Cingulate Gyrus	Intercept	0.4915	0.0195			
	Age	0.0099	0.0071	0.0068		
	Sex	0.0334	0.0119	0.0598	0.006	0.038
	Age^2	-0.0013	0.0007	0.0123		
Right Anterior Insula	Intercept	0.2476	0.0142			
	Age	0.0329	0.0054	0.1105		
	Sex	0.0152	0.0065	0.0434	0.021	0.088
	Age^2	-0.0025	0.0005	0.0705		
Left Anterior Insula	Intercept	0.2626	0.0155			
	Age	0.0331	0.0060	0.0922		
	Sex	0.0174	0.0067	0.0524	0.010	0.057
	Age^2	-0.0025	0.0006	0.0597		

Right Angular Gyrus	Intercept	1.1245	0.0432			
	Age	0.0382	0.0168	0.0161		
	Sex	-0.0140	0.0159	0.0063	0.381	0.565
	Age^2	-0.0042	0.0016	0.0214		
Left Angular Gyrus	Intercept	0.9601	0.0503			
	Age	0.0369	0.0196	0.0110		
	Sex	-0.0176	0.0180	0.0078	0.331	0.554
	Age^2	-0.0038	0.0019	0.0126		
Right Calcarine Cortex	Intercept	0.1885	0.0330			
	Age	0.0490	0.0128	0.0450		
	Sex	0.0069	0.0125	0.0026	0.583	0.767
	Age^2	-0.0051	0.0012	0.0519		
Left Calcarine Cortex	Intercept	0.3166	0.0293			
	Age	0.0202	0.0112	0.0106		
	Sex	-0.0142	0.0128	0.0102	0.268	0.516
	Age^2	-0.0023	0.0011	0.0148		
Right Central Operculum	Intercept	0.4220	0.0148			
	Age	-0.0175	0.0057	0.0304		
	Sex	-0.0003	0.0064	0.0000	0.963	0.973
	Age^2	0.0013	0.0005	0.0185		
Left Central Operculum	Intercept	0.4206	0.0138			
	Age	-0.0165	0.0053	0.0321		
	Sex	0.0069	0.0064	0.0096	0.280	0.522
	Age^2	0.0014	0.0005	0.0251		
Right Entorhinal Area	Intercept	0.0931	0.0114			
	Age	0.0175	0.0046	0.0394		
	Sex	-0.0023	0.0028	0.0067	0.408	0.589
	Age^2	-0.0015	0.0004	0.0323		
Left Entorhinal Area	Intercept	0.0995	0.0101			
	Age	0.0150	0.0040	0.0391		
	Sex	-0.0042	0.0027	0.0208	0.130	0.341
	Age^2	-0.0013	0.0004	0.0332		
Right Frontal Pole	Intercept	0.3266	0.0226			
	Age	0.0212	0.0087	0.0190		
	Sex	-0.0154	0.0095	0.0208	0.108	0.316
	Age^2	-0.0018	0.0008	0.0156		

Left Frontal Pole	Intercept	0.2615	0.0237			
	Age	0.0308	0.0092	0.0337		
	Sex	0.0090	0.0086	0.0086	0.301	0.543
	Age^2	-0.0030	0.0009	0.0346		
Right Fusiform Gyrus	Intercept	0.5372	0.0273			
	Age	0.0391	0.0105	0.0426		
	Sex	0.0128	0.0110	0.0108	0.248	0.507
	Age^2	-0.0033	0.0010	0.0326		
Left Fusiform Gyrus	Intercept	0.5645	0.0248			
	Age	0.0435	0.0094	0.0669		
	Sex	0.0079	0.0113	0.0041	0.486	0.687
	Age^2	-0.0039	0.0009	0.0579		
Right Inferior Occipital Gyrus	Intercept	0.6098	0.0417			
	Age	0.0276	0.0164	0.0087		
	Sex	0.0203	0.0136	0.0194	0.137	0.341
	Age^2	-0.0034	0.0016	0.0146		
Left Inferior Temporal Gyrus	Intercept	0.8620	0.0411			
	Age	0.0390	0.0161	0.0175		
	Sex	0.0043	0.0137	0.0008	0.754	0.836
	Age^2	-0.0033	0.0016	0.0134		
Right Lingual Gyrus	Intercept	0.8208	0.0444			
	Age	0.0237	0.0174	0.0057		
	Sex	0.0154	0.0154	0.0081	0.319	0.544
	Age^2	-0.0030	0.0017	0.0101		
Right Middle Frontal Gyrus	Intercept	2.0048	0.0661			
	Age	0.0785	0.0256	0.0298		
	Sex	0.0358	0.0262	0.0155	0.175	0.410
	Age^2	-0.0072	0.0025	0.0271		
Right Middle Occipital Gyrus	Intercept	0.6063	0.0282			
	Age	0.0035	0.0109	0.0003		
	Sex	-0.0023	0.0118	0.0003	0.843	0.891
	Age^2	-0.0015	0.0011	0.0068		
Right Postcentral Gyrus Medial Segment	Intercept	0.1690	0.0123			
	Age	-0.0222	0.0048	0.0643		
	Sex	-0.0025	0.0045	0.0027	0.57622026	0.76674575
	Age^2	0.0016	0.0005	0.0390		

Left Postcentral Gyrus Medial Segment	Intercept	0.2001	0.0159			
	Age	-0.0352	0.0063	0.0541		
	Sex	-0.0945	0.0227	0.0490	<.001	0.010
	Age^2	0.0029	0.0006	0.0333		
	Age*Sex	0.0322	0.0090	0.0393	<.001	0.035
	Age^2*Sex	-0.0029	0.0009	0.0341	0.001	0.048
Right Precentral Gyrus Medial Segment	Intercept	0.3032	0.0148			
	Age	-0.0240	0.0057	0.0540		
	Sex	0.0065	0.0058	0.0101	0.268	0.516
	Age^2	0.0018	0.0006	0.0326		
Left Precentral Gyrus Medial Segment	Intercept	0.3332	0.0132			
	Age	-0.0286	0.0051	0.0906		
	Sex	-0.0064	0.0050	0.0139	0.200	0.429
	Age^2	0.0022	0.0005	0.0609		
Right Middle Temporal Gyrus	Intercept	1.2425	0.0506			
	Age	0.0963	0.0196	0.0726		
	Sex	-0.0018	0.0201	0.0001	0.929	0.960
	Age^2	-0.0086	0.0019	0.0629		
Left Middle Temporal Gyrus	Intercept	1.3096	0.0477			
	Age	0.0639	0.0185	0.0366		
	Sex	-0.0248	0.0183	0.0147	0.178	0.410
	Age^2	-0.0060	0.0018	0.0347		
Right Orbital Part of the Inferior Frontal Gyrus	Intercept	0.1479	0.0133			
	Age	0.0090	0.0051	0.0103		
	Sex	0.0074	0.0058	0.0128	0.205	0.429
	Age^2	-0.0008	0.0005	0.0080		
Right Precuneus	Intercept	1.1152	0.0396			
	Age	0.0148	0.0152	0.0032		
	Sex	0.0001	0.0167	0.0000	0.994	0.994
	Age^2	-0.0021	0.0015	0.0070		
Left Parahippocampal Gyrus	Intercept	0.2451	0.0189			
	Age	0.0146	0.0074	0.0116		
	Sex	-0.0089	0.0059	0.0190	0.134	0.341
	Age^2	-0.0014	0.0007	0.0122		
Right Posterior Insula	Intercept	0.1182	0.0138			
	Age	0.0195	0.0052	0.0441		

	Sex	0.0205	0.0062	0.0836	0.001	0.015
	Age^2	-0.0014	0.0005	0.0260		
Left Posterior Insula	Intercept	0.1802	0.0090			
	Age	0.0126	0.0034	0.0423		
	Sex	0.0041	0.0040	0.0083	0.313	0.544
	Age^2	-0.0011	0.0003	0.0358		
Left Parietal Operculum	Intercept	0.2598	0.0123			
	Age	-0.0092	0.0045	0.0142		
	Sex	0.0111	0.0069	0.0206	0.113	0.316
	Age^2	0.0010	0.0004	0.0163		
Right Postcentral Gyrus	Intercept	1.3610	0.0603			
	Age	-0.1065	0.0239	0.0561		
	Sex	-0.0346	0.0176	0.0328	0.052	0.179
	Age^2	0.0078	0.0023	0.0333		
Left Postcentral Gyrus	Intercept	1.4311	0.0724			
	Age	-0.0711	0.0288	0.0175		
	Sex	-0.0607	0.0198	0.0781	0.003	0.021
	Age^2	0.0044	0.0028	0.0072		
Right Posterior Orbital Gyrus	Intercept	0.2493	0.0133			
	Age	0.0080	0.0051	0.0081		
	Sex	0.0059	0.0059	0.0083	0.315	0.544
	Age^2	-0.0009	0.0005	0.0116		
Left Posterior Orbital Gyrus	Intercept	0.2659	0.0140			
	Age	0.0089	0.0053	0.0096		
	Sex	0.0036	0.0071	0.0021	0.613	0.778
	Age^2	-0.0009	0.0005	0.0115		
Right Planum Polare	Intercept	0.1584	0.0105			
	Age	0.0108	0.0042	0.0204		
	Sex	-0.0017	0.0034	0.0021	0.626	0.778
	Age^2	-0.0011	0.0004	0.0215		
Right Subcallosal Area	Intercept	0.0973	0.0079			
	Age	0.0040	0.0031	0.0053		
	Sex	0.0061	0.0030	0.0332	0.043	0.166
	Age^2	-0.0005	0.0003	0.0080		
	Intercept	0.5236	0.0246			
	Age	-0.0250	0.0096	0.0202		

Right Supplementary Motor Cortex	Sex	-0.0038	0.0081	0.0018	0.643	0.778
	Age^2	0.0020	0.0009	0.0139		
Right Supramarginal Gyrus	Intercept	0.7916	0.0442			
	Age	0.0525	0.0169	0.0311		
	Sex	-0.0343	0.0192	0.0262	0.077	0.254
	Age^2	-0.0055	0.0016	0.0367		
Right Superior Occipital Gyrus	Intercept	0.4036	0.0302			
	Age	0.0167	0.0119	0.0061		
	Sex	-0.0042	0.0100	0.0015	0.678	0.789
	Age^2	-0.0021	0.0012	0.0100		
Right Superior Parietal Lobule	Intercept	1.3066	0.0805			
	Age	-0.0822	0.0321	0.0184		
	Sex	0.0068	0.0216	0.0008	0.752	0.836
	Age^2	0.0053	0.0031	0.0083		
Right Superior Temporal Gyrus	Intercept	0.7336	0.0318			
	Age	0.0144	0.0122	0.0047		
	Sex	0.0127	0.0139	0.0070	0.364	0.559
	Age^2	-0.0020	0.0012	0.0093		
Left Superior Temporal Gyrus	Intercept	0.6392	0.0358			
	Age	0.0426	0.0140	0.0283		
	Sex	0.0055	0.0124	0.0017	0.658	0.783
	Age^2	-0.0042	0.0014	0.0291		
Right Temporal Pole	Intercept	0.4796	0.0310			
	Age	0.0642	0.0120	0.0854		
	Sex	0.0011	0.0125	0.0001	0.931	0.960
	Age^2	-0.0051	0.0012	0.0592		
Left Temporal Pole	Intercept	0.4664	0.0344			
	Age	0.0566	0.0134	0.0535		
	Sex	0.0187	0.0128	0.0169	0.145	0.352
	Age^2	-0.0043	0.0013	0.0343		
Right Triangular Part of the inferior frontal gyrus	Intercept	0.2866	0.0240			
	Age	0.0466	0.0094	0.0728		
	Sex	-0.0041	0.0081	0.0024	0.616	0.974
	Age^2	-0.0045	0.0009	0.0741		
Right Transverse Temporal Gyrus	Intercept	0.1712	0.0099			
	Age	-0.0102	0.0038	0.0236		

	Sex	0.0049	0.0046	0.0094	0.284	0.522
	Age^2	0.0006	0.0004	0.0106		
Left Transverse Temporal Gyrus	Intercept	0.1768	0.0083			
	Age	-0.0078	0.0029	0.0241		
	Sex	0.0090	0.0056	0.0202	0.113	0.316
	Age^2	0.0006	0.0003	0.0167		
Right Caudate	Intercept	0.3454	0.0045			
	Age	-0.0026	0.0006	0.1185		
	Sex	-0.0262	0.0064	0.0594	<.001	0.006
	Age*Sex	0.0001	0.0008	0.0001	0.875	0.969
Right Pallidum	Intercept	0.0930	0.1959			
	Age	0.0002	0.0339	0.0166		
	Sex	-0.0092	0.2714	0.0289	0.001	0.038
	Age*Sex	0.0007	0.0475	0.0062	0.161	0.869
Right Ventral Diencephalon	Intercept	0.3065	0.0036			
	Age	0.0053	0.0005	0.3174		
	Sex	-0.0122	0.0040	0.0729	0.003	0.021
Left Basal Forebrain	Intercept	0.0672	0.0014			
	Age	-0.0011	0.0002	0.0555		
	Sex	0.0024	0.0010	0.0418	0.027	0.106
Right Basal Forebrain	Intercept	0.0654	0.0012			
	Age	-0.0011	0.0002	0.0806		
	Sex	0.0012	0.0009	0.0158	0.183	0.411
Right Anterior Orbital Gyrus	Intercept	0.2214	0.0056			
	Age	-0.0025	0.0009	0.0258		
	Sex	-0.0025	0.0052	0.0018	0.631	0.778
Left Anterior Orbital Gyrus	Intercept	0.1888	0.0043			
	Age	-0.0015	0.0007	0.0153		
	Sex	-0.0070	0.0040	0.0250	0.081	0.256
Right Cuneus	Intercept	0.5382	0.0102			
	Age	-0.0119	0.0013	0.2167		
	Sex	0.0052	0.0112	0.0018	0.640	0.778
Left Cuneus	Intercept	0.5331	0.0101			
	Age	-0.0135	0.0014	0.2510		
	Sex	0.0104	0.0110	0.0075	0.346	0.559
Right Gyrus Rectus	Intercept	0.1752	0.0065			
	Age	0.0018	0.0011	0.0072		

	Sex	-0.0055	0.0049	0.0110	0.269	0.516
Left Gyrus Rectus	Intercept	0.1742	0.0057			
	Age	0.0019	0.0010	0.0103		
	Sex	-0.0125	0.0042	0.0741	0.003	0.025
Left Inferior Occipital Gyrus	Intercept	0.7021	0.0162			
	Age	-0.0099	0.0026	0.0400		
	Sex	0.0560	0.0142	0.1089	<.001	0.002
Left Lingual Gyrus	Intercept	0.8300	0.0140			
	Age	-0.0046	0.0022	0.0129		
	Sex	-0.0117	0.0127	0.0071	0.359	0.559
Left Lateral Orbital Gyrus	Intercept	0.2441	0.0068			
	Age	-0.0035	0.0010	0.0358		
	Sex	0.0024	0.0066	0.0012	0.711	0.818
Right Middle Cingulate Gyrus	Intercept	0.4581	0.0078			
	Age	-0.0053	0.0012	0.0588		
	Sex	0.0062	0.0075	0.0057	0.409	0.589
Left Middle Cingulate Gyrus	Intercept	0.4920	0.0103			
	Age	-0.0110	0.0017	0.1194		
	Sex	0.0289	0.0092	0.0796	0.002	0.019
Left Middle Occipital Gyrus	Intercept	0.6268	0.0121			
	Age	-0.0105	0.0018	0.1040		
	Sex	0.0115	0.0122	0.0071	0.346	0.559
Right Superior Frontal Gyrus Medial Segment	Intercept	0.7737	0.0131			
	Age	-0.0043	0.0018	0.0174		
	Sex	0.0060	0.0137	0.0015	0.664	0.783
Left Occipital Pole	Intercept	0.1611	0.0139			
	Age	0.0066	0.0022	0.0253		
	Sex	0.0108	0.0121	0.0066	0.374	0.564
Right Occipital Fusiform Gyrus	Intercept	0.5122	0.0123			
	Age	-0.0166	0.0023	0.1261		
	Sex	-0.0446	0.0169	0.0173	0.009	0.183
	Age*Sex	0.0110	0.0032	0.0341	0.001	0.037
Left Occipital Fusiform Gyrus	Intercept	0.4852	0.0096			
	Age	-0.0110	0.0016	0.1196		
	Sex	0.0073	0.0080	0.0068	0.364	0.559
Left Posterior Cingulate Gyrus	Intercept	0.4859	0.0072			
	Age	-0.0044	0.0010	0.0676		

	Sex	0.0208	0.0078	0.0566	0.008	0.051
Left Precuneus	Intercept	1.2273	0.0157			
	Age	-0.0153	0.0022	0.1425		
	Sex	-0.0013	0.0166	0.0000	0.939	0.960
Right Precentral Gyrus	Intercept	1.2174	0.0171			
	Age	-0.0166	0.0028	0.0980		
	Sex	0.0229	0.0150	0.0190	0.130	0.341
Left Precentral Gyrus	Intercept	1.2002	0.0183			
	Age	-0.0108	0.0029	0.0404		
	Sex	-0.0094	0.0165	0.0027	0.572	0.767
Right Planum Temporale	Intercept	0.1869	0.0047			
	Age	-0.0016	0.0007	0.0182		
	Sex	0.0010	0.0049	0.0003	0.841	0.891
Left Planum Temporale	Intercept	0.2085	0.0050			
	Age	-0.0017	0.0007	0.0199		
	Sex	0.0107	0.0054	0.0317	0.047	0.173
Left Superior Occipital Gyrus	Intercept	0.4293	0.0112			
	Age	-0.0060	0.0017	0.0364		
	Sex	-0.0036	0.0105	0.0010	0.730	0.829
Left Superior Parietal Lobule	Intercept	1.1780	0.0260			
	Age	-0.0216	0.0044	0.0657		
	Sex	-0.0276	0.0214	0.0135	0.200	0.429
Left Triangular Part of the Inferior Frontal Gyrus	Intercept	0.4295	0.0098			
	Age	0.0020	0.0014	0.0060		
	Sex	-0.0229	0.0097	0.0437	0.021	0.088
